# Fruit‐Based Diet and Gut Health: A Review

**DOI:** 10.1002/fsn3.70159

**Published:** 2025-04-30

**Authors:** Sammra Maqsood, Muhammad Tayyab Arshad, Ali Ikram, Kodjo Théodore Gnedeka

**Affiliations:** ^1^ National Institute of Food Science and Technology University of Agriculture Faisalabad Faisalabad Pakistan; ^2^ University Institute of Food Science and Technology The University of Lahore Lahore Pakistan; ^3^ Togo Laboratory: Applied Agricultural Economics Research Team (ERE2A) University of Lomé Lomé Togo

**Keywords:** antioxidant, gut microbiota, inflammation, prebiotic

## Abstract

Gut health is essential to the overall well‐being of a human being due to its implication on digestion, the performance of the immune system, and nutritional absorption. The gut microbiota represents an intricate ecology of bacteria, fungi, and viruses, important in regulating the immune response and maintaining intestinal health. Fruit‐based diets have developed as an essential constituent in gut health, and current studies highlight nutrition in modulating gut microbiota composition and activity. Rich in fiber, polyphenols, vitamins, and antioxidants, fruits also expand immunological function, subordinate inflammation in the stomach, and boost microbial diversity. The article reviews the benefits of fruit‐derived dietary fibers, which assist as prebiotics in fostering the development of beneficial gut microbiota and decreasing intestinal inflammation. These antioxidants in fruits include flavonoids and carotenoids, whose immunomodulatory properties are under investigation for therapeutic use in autoimmune diseases, infections, and inflammatory bowel disease (IBD). Some fruits of particular interest include bananas, apples, citrus, and berries, as studies have consistently shown their immunomodulatory and gastrointestinal effects. There are still barriers to increasing fruit intake, including socioeconomic restrictions and the need for personalized nutritional counseling. The review fills an existing gap in the literature. It encourages enhanced immune and gastrointestinal well‐being by combining the most recent research with practical recommendations on implementing fruit‐based diets into daily nutrition.

## Graphical abstract

Gut health is integral to well‐being, influencing digestion, immune responses, and nutrient absorption. Our work synthesizes current research on the contributions of fruit‐derived dietary fibers and polyphenols to gut microbial diversity and reduced inflammation, emphasizing their prebiotic properties and therapeutic potential. We also explore the immune‐modulatory effects of antioxidants and specific fruits, including berries, citrus fruits, apples, and bananas, which have shown pronounced benefits in gut health and immunity. Additionally, the manuscript addresses challenges to fruit consumption and the importance of personalized dietary counseling, aiming to bridge gaps in knowledge and practice.

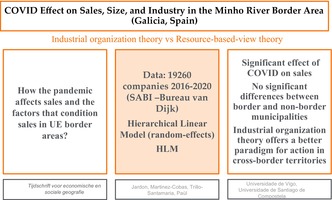

**Abbreviations**
CKD: Chronic Kidney Disease
CRP: C‐Reactive Protein
FODMAPs: fermentable oligosaccharides, disaccharides, monosaccharides, and polyols
FOS: Fructooligosaccharides
GALT: Gut‐Associated Lymphoid Tissue
IBD: Inflammatory Bowel Disease
IL‐6: Interleukin 6
NF‐κB: Nuclear Factor Kappa‐light‐chain‐enhancer of activated B‐cells
ROS: Reactive Oxygen Species
ROS: Reactive Oxygen Species
SCFAs: Short‐Chain Fatty Acids
T‐cells: T‐lymphocytes
TNF‐α: Tumor Necrosis Factor Alpha

## 1 Introduction

The gut microbiome, encompassing trillions of microorganisms in the gastrointestinal system, is vital to human health since it regulates the immunological reaction, metabolic mechanism, and digestion (Afzaal et al. 2022). Due to their abundance of fiber and polyphenols, fruits endorse a balanced microbiota, and hence they have a noteworthy positive effect on gut health (Henning et al. 2017). As prebiotics, dietary fibers augment the integrity of the gut barrier by acting as substrates for good gut bacteria, which inspires their proliferation and improves the production of short‐chain fatty acids (SCFAs) (Beukema et al. 2020; Ikram et al. 2024; Dreher 2018). Fruits high in fiber and polyphenols, such as berries and citrus fruits, have antibacterial properties and modulate gut microbial variety, plummeting inflammation and benefiting systemic health (Bouyahya et al. 2022; Alonso and Guarner 2013). Fruits rich in fiber and polyphenols assist a healthy gut flora and diminish the chance of inflammatory and metabolic diseases (Elshahed et al. 2021; Henning et al. 2017).

The human gut is more than a mere digestive organ because it harbors trillions of microorganisms collectively called the gut microbiota (Dong et al. 2025; Beukema et al. 2020). This collective entity is diverse and ever‐changing, comprising fungi, viruses, bacteria, and archaea (Rist et al. 2013). The gut microbiota is crucial for maintaining an organism's overall health because it controls physiological processes such as immune response, nutrition absorption, and digestion (Wan et al. 2019). The complex interaction of gut microbiota with human health has been a concern of researchers, who highlighted that retaining homeostasis and averting illness is essential. The gut bacteria facilitate the digestive process because it helps digest the complex portions of foodstuffs that the human body cannot digest on its own. Two examples include fiber and polysaccharides (Aziz et al. 2013). These fermented substrates produce SCFAs, such as butyrate, propionate, and acetate (Fouhse et al. 2016). SCFAs diminish inflammation and enhance glucose metabolism, providing energy to colonocytes and improving systemic health (Pluske et al. 2018; Abd El‐Aziz et al. 2024). Other essential vitamins, such as vitamin K and specific B vitamins, are produced by gut bacteria and are involved in food digestion and the body's general health (Kogut et al. 2017).

The gut microbiota plays a key role in the immune system and aids digestion. The maturation of the immune system and its developmental stages depend on this process, especially during the early periods of life when microbial colonization establishes immunological homeostasis (Ikram et al. 2021; Rajoka et al. 2017). The microbiota communicates with the host immune system through microbial metabolites and pattern recognition receptors, such as Toll‐like receptors, capable of recognizing MAMPs (Chen et al. 2023). This interaction modifies immunological responses, increases the production of regulatory T‐cells, and lowers the risk of chronic inflammation, according to Hollister et al. (2014). Gut health also includes the integrity of the gut barrier. The entry of toxins and other pathogens into systemic circulation cannot occur when the gut barrier is intact (Xiong et al. 2022). The gut microbiota fortifies this barrier by inducing the production of mucus and tight junction proteins that seal the epithelial lining (Greenhalgh et al. 2016). However, dysbiosis or gut microbiota imbalance may lead to disorders like leaky gut syndrome, autoimmune disease, and systemic inflammation because barrier functions are affected (Yan et al. 2025; Rist et al. 2013). Dysbiosis has been associated with various diseases, including diabetes, obesity, inflammatory bowel disease (IBD), and neurodegenerative diseases (Alonso and Guarner 2013). Many factors trigger dysbiosis, including poor dietary intake, the use of antibiotics, and chronic stress. Diet impacts the gut microbiota. Diets with a higher content of plant‐based fiber, polyphenols, and fermented food components support a more diverse community of microorganisms and their growth. Still, diets heavy in processed food and saturated fat have opposite effects (Das and Nair 2019). One of the main aims of preventive medicine is improving gut health through dietary and lifestyle changes. Probiotics are live beneficial bacteria, while prebiotics are non‐digestible food elements that support beneficial microbes and have been increasingly recognized to improve immune function and restore microbial balance (Vitetta et al. 2014). The gut microbiota could be an attractive target for therapy to improve health outcomes due to its flexibility and robustness (Abbas et al. 2022; Bäckhed et al. 2012).

This review emphasizes the importance of fruit‐based diets for enhancing immunological and gastrointestinal health. Fruits boost the gut microbiota environment for efficient digestion and nutrient absorption and balance the immune response by promoting microbial diversity and providing vital nutrients such as fibers, antioxidants, and polyphenols. The consequences, therefore, underline fruits as a relatively cheap, readily available diet for managing and preventing chronic inflammation, autoimmune diseases, and gastrointestinal conditions. Further, the practical advice on fruit intake provides achievable guidance to individuals who intend to utilize their diet to enhance their health.

With the growing new evidence highlighting the intricate interrelationship among nutrition, the microbiome, and immunological regulation, the studies on fruit‐based diets and their influences on gut health and immune system functions are attaining greater and greater significance. While there is an ever‐cumulative corpus of data, thorough assessments are still missing, precisely examining the additive properties of numerous fruits on gut health and immunological function. This comprehensive review aims to close the aforementioned research gap by integrating the most current data on how different fruits, each with a different nutritional profile, impact gut microbial diversity, inflammation levels, and immune responses. The significance of this review lies in the fact that it makes gut health improvements and immune‐related disorders in the potential offspring through clear, evidence‐based consumption advice for fruits, leading to straight clinical and public health arrangements.

## 2 Gut Microbiota Composition: Microorganisms in the Gut

**FIGURE 1:**
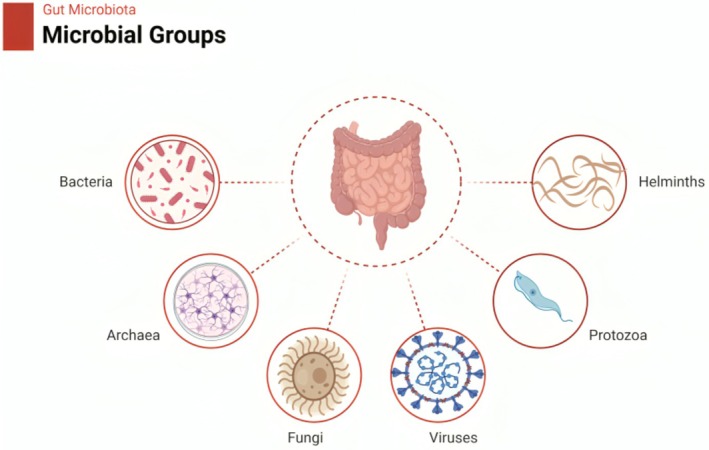
Microbes in the gut.

“Gut microbiota” is the complex and diverse community of bacteria, fungi, viruses, and archaea that live in the human gut (Figure 1). These microbes peacefully coexist with their host and are essential for digestion, immune function, nutrient absorption, and general health (Afzaal et al. 2022). The most common bacterial species are *Firmicutes* and *Bacteroidetes*, while *Actinobacteria*, *Proteobacteria*, and *Verrucomicrobia* are less common. Most of the viral population consists of bacteriophages, which influence bacterial dynamics, whereas fungi are less well studied and include species such as *Candida* (Henning et al. 2017). Understanding the diversity of microbes is vital since each one is different in its ability to promote host health or cause disease. For example, bacteria degrade dietary fibers into SCFAs, which allow the immune system to regulate gut integrity (Das and Nair 2019). Imbalance is referred to as this condition, and it brings about a range of health concerns, such as infections, metabolic disorders, and even IBD (Fouhse et al. 2016).

Diversity and gut microbiota composition are among the most critical elements that control human health (Greenhalgh et al. 2016). Generally, a diverse microbiome is associated with improved metabolic and immunological function and infection resistance. Environmental, genetic, antibiotic, and nutritional factors impact this type (Assimakopoulos et al. 2018). A high microbial diversity system that responds well to environmental changes is associated with ecological stability, which includes an intact gut barrier and minor harmful inflammation (Aziz et al. 2013). Conversely, a lower level of diversity is associated with a higher risk of obesity, diabetes, and cardiovascular diseases. Research has shown that diets rich in fiber and polyphenols promote the growth of beneficial microbes and improve microbial diversity, whereas diets rich in fat and sugar promote the development of pathogenic species and induce inflammation (Cénit et al. 2014). Microbial imbalance is associated with diseases seemingly unconnected with the gut. These can include diseases relating to liver disorders by a gut–liver axis and the neurological system through neuropsychiatric diseases through a gut–brain axis (Zhu et al. 2022). Proof of the systemic implication of microbiota imbalance results from the fact that several products manufactured within the Microbiota system have been tested to cause neural health and traverse the blood–brain barrier (Sherwin et al. 2018). The diversity and composition of the gut microbiota are vital markers of a healthy community and are essential for maintaining health. Diet and lifestyle interventions that favor microbial diversity can prevent and treat a broad spectrum of disorders. However, to fully exploit the potential of microbiota as a major therapeutic modality, more research must be directed toward targeted regulation of this entity.

Gut dysbiosis is the imbalanced state of normal gut microbiota, leading to various disorders that severely damage health (Jhee et al. 2019). The multiple microbes—such as bacteria, fungi, viruses, and all other kinds—form the “gut microbiome”; they play an essential role in keeping the immunological flora balanced (Fouhse et al. 2016). Dysbiosis upsets this balance, causing a chain of pathological alterations that may be expressed as IBDs, autoimmune diseases, and infections (Sherwin et al. 2018). The role of gut homeostatic balance in immunity is demonstrated by autoimmune diseases and gut microbiota. Imbalanced microbial diversity leads to exaggerated immunity, which enhances susceptibility to autoimmune diseases like type 1 diabetes and rheumatoid arthritis. According to Cénit et al. (2014), some imbalances in microbes have a synergetic effect on autoimmunity, causing tissue death and chronic inflammation due to the increased production of pro‐inflammatory cytokines. Crohn's disease and ulcerative colitis are two IBDs strongly associated with gut dysbiosis. Alterations in the composition of gut microbes promote the proliferation of pathogenic species while reducing the abundance of *Lactobacillus* and *Bifidobacterium*, two good bacteria (Cénit et al. 2014). This disruption weakens the gut barrier and opens avenues through the intestinal mucosa, giving poisonous chemicals and harmful microbes a chance to infiltrate and trigger immunologic reactions (Assimakopoulos et al. 2018). According to Fouhse, Zijlstra, and Willing, such interactions trigger chronic inflammation, a signature characteristic of IBD (Fouhse et al. 2016).

Additionally, dysbiosis increases the susceptibility to the disease. The loss of helpful microbial species impairs the gut's ability to outcompete dangerous bacteria. For example, the overproduction of *Clostridium difficile* leads to severe diarrhea and colitis in dysbiotic gut disorders. To prevent opportunistic pathogen invasion, von Martels et al. (2017) emphasize maintaining balance in the bacteria.

## 3 Diet Importance in Gut Health

One factor that has been proven to influence the composition and activities of gut microbiota the most is diet (Fouhse et al. 2016). A rich and diverse microbial community has been proven to be promoted by a diet rich in fruit, fiber, polyphenols, and natural sugars (Das and Nair 2019). Fibers serve as a substrate for gut bacteria, which ferment to produce SCFAs with anti‐inflammatory effects on the integrity of the gut barrier (Pluske et al. 2018). According to Wan et al. (2019), fruits are rich in polyphenol concentrations, which are known to have prebiotic effects and encourage the growth of beneficial bacteria, including *Bifidobacteria* and *Lactobacilli*. In addition, fruits significantly affect immune function modulation and gastrointestinal health (El‐Sabrout et al. 2024). In fruits, antioxidants and vitamins, including vitamin C, reduce gastrointestinal inflammation and oxidative stress, strengthening the immune system (Bai et al. 2024; Rajoka et al. 2017).

A healthy gut also contributes to more robust immune responses, and diets rich in fruits have been associated with higher microbial diversity (Aziz et al. 2013). This results in dysbiosis. An imbalance of gut flora due to an excessive intake of processed food, saturated fats, and low intake of fiber has been identified to cause various issues related to immunological function, including disruption of the intestinal barrier, systemic inflammation, and SCFA synthesis impairments (Rist et al. 2013). Besides improving the gut's health, fruit‐based diets would reduce the occurrence of chronic diseases such as diabetes, cardiovascular disease, and inflammatory disorders that can be potentially detrimental to health outcomes (Das and Nair 2019). With the connection between the immune system and the stomach, one has to take care of his diet if he desires gut health to stay healthy. Much of human health is contingent on the assembly of the immune system and the stomach, and novel research specifies a diet's critical role in keeping the gut healthy (El‐Sabrout et al. 2024). Since the gut microbiota composition directly impacts the growth and function of the immune system, it is very significant for regulating immunological responses (Alonso and Guarner 2013). In this respect, fruits are especially beneficial because of their fiber and bioactive compounds, which provide gastrointestinal health and immunity and endorse gut bacteria. For instance, flavonoids, polyphenols, and fibers have been established to exert prebiotic properties by promoting the development of beneficial flora that enhance intestinal motility and modulate immune function (Katsirma et al. 2021).

On the other hand, regular intake of fruits is also related to improved metabolic profiles, which help decrease the morbidity of chronic diseases and modulate inflammation due to the effect on lipid profiles, insulin resistance, and liver biomarkers (Alami et al. 2022). Besides preventing dysbiosis and associated diseases, it maintains gut barrier integrity, which is achieved through a balanced diet rich in fruits (Beigoli and Boskabady 2024). Diet not only provides energy for the body but also plays a crucial role in immunological regulation; thus, the requirement of comprising a range of fruits in one's diet to keep a healthy gut‐immune system axis is emphasized (Aziz et al. 2013; Hooper et al. 2012). This particular dietary option ensures the consumption of nutrients and substrates in fruit‐based diets to promote gut flora, thus protecting the body from diseases linked to immunities. It demonstrates the massive impact nutritional factors may have on health by seeming to be more like an introduction to preventing several diseases.

## 4 Nutritional Components in Fruits

### 4.1 Fibers

Fruit fibers are mainly categorized as soluble and insoluble; both have different roles to play in maintaining intestinal health. The soluble fiber in fruits like apples, citrus, and berries dissolves in water and forms a gel, enhancing good gut flora growth (Rist et al. 2013). It produces SCFAs that are butyrate, acetate, and propionate, which help regulate immunity and gut health (Henning et al. 2017). Soluble fiber has prebiotic potential, which favors a balanced gut microbiota and microbial diversity (Oliveira et al. 2018). These insoluble fibers in fruits, such as pears and prunes, remain intact during the digestive process. They help enhance intestinal motility through bowel movements and prevent constipation (Septembre‐Malaterre et al. 2018). Together, these fibers strengthen the gut barrier, prevent harmful microbes from entering it, and reduce intestinal inflammation (Jaiswal 2020).

### 4.2 Polyphenols and Flavonoids

It has been well‐documented that pomegranates, cherries, and blueberries are rich in flavonoids and polyphenols, which strongly affect the makeup of gut flora (Jaiswal 2020). It is well established that polyphenols inhibit the proliferation of pathogens while stimulating beneficial ones, *Lactobacillus* and *Bifidobacterium* (Lebaka et al. 2021). This shift in the microbiome may reduce dysbiosis, an imbalance in the microbes, a condition associated with chronic diseases such as IBD and obesity (Komati et al. 2024a, 2024b). flavonoids, a subclass of polyphenols, have shown anti‐inflammatory characteristics by inhibiting pro‐inflammatory cytokines such as TNF‐α and IL‐6 (Alami et al. 2022). Moreover, these compounds function as antioxidants, scavenging free radicals and, thus, preventing oxidative cell damage in the gastrointestinal area (Maldonado‐Celis et al. 2019).

### 4.3 Vitamins, Minerals, and Antioxidants

Fruits are the best source of vitamins and antioxidants, significantly impacting intestinal health (Gâtlan and Gutt 2021). Vitamin C, found in citrus fruits, kiwis, and strawberries, helps fortify the epithelial integrity of the gut lining and protects against oxidative stress from ROS, reducing damage (Miles and Calder 2021). Vitamin E, a fruit like mango and avocado, protects lipids within the cell membrane of the gastrointestinal epithelial cells from oxidative damage (Vincente et al. 2014). Many fruits, such as cherries and grapes, contain antioxidants like carotenoids and anthocyanins. These compounds reduce oxidative stress, which heals damaged cells and reduces inflammation in the gut (Gâtlan and Gutt 2021). Fruits are a source of vitamins and antioxidants that synergistically mitigate the risk of chronic diseases and support healthy microbial ecosystems in the gut (Crowe‐White et al. 2016). Table 1 describes nutritional components and their impact on gut health.

**TABLE 1:** Nutritional components of fruits and their impact on gut health.

Component	Impact	Reference
Fibers (Soluble)	Promotes gut health by supporting beneficial bacteria and producing SCFAs like butyrate, which helps reduce inflammation	Slavin (2013)
Fibers (Insoluble)	Aids in bowel regularity, increases stool bulk, and supports overall gut motility	Meyer et al. (2020)
Polyphenols	Modulates microbiota composition, promoting beneficial bacteria such as *Lactobacillus* and *Bifidobacterium*.	Salehi et al. (2019)
Flavonoids	Exhibits antioxidant and anti‐inflammatory effects, promoting a healthy gut microbiome	Bohn et al. (2024)
Vitamin C	Reduces oxidative stress, gut inflammation, and supports immune function in the gut	Fazio et al. (2021)
Vitamin E	Provides antioxidant protection, reducing gut inflammation and oxidative stress	Ziegler et al. (2021)
Probiotics	Strengthens the gut microbiota by providing live beneficial bacteria, promoting gut health and digestion	de Souza et al. (2019)
Prebiotics	Fosters the growth of beneficial bacteria like *bifidobacteria* and lactobacilli, which enhance gut health and immune function	Gibson et al. (2017)
Magnesium	Plays a role in maintaining gut motility and relieving constipation, supporting muscle relaxation in the digestive tract	Wallace et al. (2019)
Potassium	Helps in regulating gut function by balancing fluid and electrolyte levels in the digestive system	Aburto et al. (2013)
Carotenoids	Antioxidant‐rich compounds (e.g., beta‐carotene) that help reduce gut inflammation and support immune health	Tan et al. (2014)
Folic Acid	Supports the repair and regeneration of gut lining cells and plays a role in reducing inflammation	Ikram et al. (2024)
Lutein	Reduces oxidative stress in the gut and supports healthy digestive tract function	Ma et al. (2020)
Tannins	Have anti‐inflammatory and antioxidant properties that help modulate gut microbiota and reduce gut inflammation	Totaro et al. (2023)

Besides the vitamins and antioxidants, fruits have many essential minerals that help gut health. These minerals are vital for preserving a stable microbiota and the healthy procedure of the gastrointestinal tract (Gâtlan and Gutt 2021). For example, potassium, which is found in high amounts in fruits such as bananas, oranges, and melons, helps maintain fluid balance and prevent constipation by encouraging regular bowel movements (Dreher 2018). Moreover, it has been shown that magnesium, also found in fruits such as avocado and fig, promotes the functioning of smooth muscles by enhancing intestinal motility, thus lessening one's discomfort or bloating (Hunter et al. 2016). In addition, folate, an essential B vitamin for DNA synthesis and reparation, is present in large quantities in citrus fruits and berries. This vitamin is necessary for maintaining gut cell reliability and endorsing the best possible intestinal function (Liu 2013; Tufail et al. 2024).

Furthermore, folate has been shown to ease the growth of advantageous gut bacteria, particularly those that degrade SCFAs, which are crucial for upholding gut integrity and reducing inflammation (Komati et al. 2024a, 2024b). Lastly, copper in fruits such as avocados is crucial for sustaining the intestinal epithelial barrier and iron absorption, which helps prevent leaky gut syndrome (Maugeri et al. 2019). A variety of fruits added to the diet can help optimize the microbiota's function in addition to supplying these essential minerals. Soluble fibers, taken from fruit in the diet, for instance, including pectin from apples and citrus fruits, are linked to the proliferation of beneficial gut microbiota such as *Lactobacilli* and *Bifidobacteria* responsible for the delicate balance of an immune system with a healthy gut (de Andra et al. 2021; Elshahed et al. 2021). Fruits indirectly stimulate immunological responses, improve digestive competence, and decrease systemic inflammation by replenishing the gut microbiome with these helpful microbes (Fouhse et al. 2016).

### 4.4 Gut Health and Nutrient Interaction

The interaction of fibers, polyphenols, flavonoids, vitamins, and antioxidants further fosters a symbiotic relationship between gut microbiota and diet (Stuhl et al. 2011). Fruit‐based diets have been proven to lessen intestinal inflammation, increase bacterial populations that produce SCFAs, and promote diversity in microbes (Olazcuaga et al. 2023). This would have wide‐ranging implications for preventing disorders such as non‐alcoholic fatty liver disease, type 2 diabetes, and cardiovascular conditions (Satija and Hu 2018; Stuhl et al. 2011). Additionally, by enhancing the mucosal immunity of the gut mucosa, these diet constituents have reduced vulnerability to the attacks of autoimmunity and pathogenic microbes against the host body (Wei et al. 2023). More dramatically noticeable is the protective impact, for instance, of this factor in diets providing several fruits that could guarantee bioactive range consumption (Komati et al. 2024a, 2024b). Because fruit‐based diets contain essential fibers, polyphenols, flavonoids, vitamins, and antioxidants, they significantly and positively affect gut health. The elements fight inflammation, reduce oxidative stress, and help maintain a balanced gut microbiome. Besides promoting digestive health, a diet of many fruits prevents chronic diseases (Satija and Hu 2018).

Humans' gut health depends on the diverse bacterial population of their gut microbiota. Diet plays a vital role in shaping this microbiome. Fruit‐rich diets are significant as a source of microbial variety (Satija and Hu 2018). Fruits are excessive substrates for gut bacteria growth and bustle since they are rich in vitamins, minerals, dietary fiber, and polyphenols. Intake of fruit variety and food diversity leads to a rich gut microenvironment that extends the ranges of nutrients and other bioactive agents (Zhang et al. 2024). High ingestion of fruits contributes to a broader array of bacterial populations, but according to scientists, it induces a healthy advanced gut microbiota (Henning et al. 2017).

## 5 Specific Fruits and Their Prebiotic Effects

**FIGURE 2:**
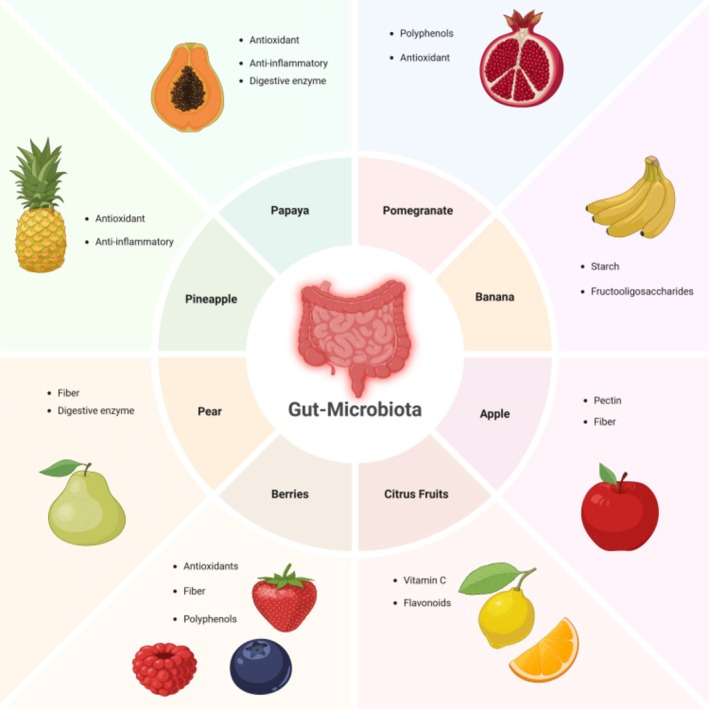
Fruits and their effects on gut health.

Because of their prebiotic properties, some fruits are more beneficial to the gut than others. Prebiotics are undigested food components that selectively stimulate the growth and activity of beneficial gut flora. Some of the following fruits have been shown to exhibit notable prebiotic effects: (1) Apples are high in soluble fiber pectin and stimulate the growth of *Lactobacilli* and *Bifidobacteria*. These microorganisms generate butyrate and other SCFAs, which reduce inflammation and maintain the gut barrier's integrity (Oliveira et al. 2018). (2) Bananas promote the growth of *Bifidobacteria* because they contain fructooligosaccharides (FOS) and resistant starch. They also support general digestive health and help control bowel movements (Vincente et al. 2014). (3) Berries are lavish in dietary fiber and polyphenols, the most notable examples being blueberries, raspberries, and strawberries. A study by Maldonado‐Celis et al. (2019) observed that polyphenols support such beneficial strains of bacteria as *Akkermansia muciniphila* . This bacterium is known for conserving the integrity of the gut barrier. Polyphenols have antibacterial activity that is contrary to damaging bacteria. (4) Citrus fruits: Vitamin C and flavonoids are present in grapefruits, oranges, and lemons. Such compounds help decrease gut inflammation by encouraging good bacteria growth and modulating immunological responses (Figure 2) (Miles and Calder 2021).

Prebiotic fruits are subjected to breakage by the gut flora, which creates the release of SCFAs, which include butyrate, propionate, and acetate (Olazcuaga et al. 2023). SCFAs endow energy on colonocytes while maintaining pH balance in the gut environment and eliminating harmful bacteria overgrowth (Liu et al. 2025). Polyphenol, conversely, refers to the biochemical byproduct from the enzymatic modification of fruit components through their microbiota transformation (Lakshmanan et al. 2022). According to Henning et al. (2017), high fruit and vegetable juice diet composition in the gut microbiota significantly changed and increased beneficial bacteria (Henning et al. 2017). Similar results were obtained by Maldonado‐Celis et al. (2019), who reported that mangos' polyphenols inhibited detrimental bacterial strains but stimulated the growth of beneficial bacteria. Consuming various fruits contributes to the diversity and functionality of gut bacteria (Maldonado‐Celis et al. 2019). Diets with high consumption of fruits help support the development of balanced gut flora. Some prebiotic fruits, including citrus, berries, bananas, and apples, aid in good gut flora formation. Therefore, a diet that includes a variety of fruits can improve gut health and reduce the risk of chronic diseases.

The breakdown of fruit dietary fibers is necessary for the gut microbiota to synthesize SCFAs. Butyrate, propionate, and acetate are the SCFAs, which are some of the essential nutrients supporting gut health and systemic immunological functions (Lakshmanan et al. 2022). The human enzymes cannot break down fruit dietary fibers that are primarily pectin, cellulose, and hemicellulose; however, they can be easily fermented in the colonic bacteria to form SCFAs (Henning et al. 2017; Olazcuaga et al. 2023). Lepaus et al. (2023) noted that this process occurs mainly within the colon, where anaerobic bacteria such as *Firmicutes* and *Bacteroidetes* predominate and effectively ferment these fibers (Olazcuaga et al. 2023). These SCFAs have been known to maintain gut integrity by strengthening the epithelial barrier and reducing intestinal permeability. For example, butyrate is the primary energy source for colonocytes; thus, this reduces oxidative stress and assists in cellular repair (Oliveira et al. 2018). SCFAs also prevent the spreading of dangerous chemicals and infections by regulating tight junction proteins like occludins and claudins (Maldonado‐Celis et al. 2019). Propionate and acetate, being anti‐inflammatory, also get into the blood. In addition, they play a role in glucose and lipid metabolism (Vincente et al. 2014). Prebiotic fibers, higher in fruits such as apples, bananas, and citrus, help promote the growth of beneficial bacteria like *Bifidobacteria* and *Lactobacilli*. High fruit consumption has been associated with higher microbial diversity and SCFA production, as reported by studies (Meena et al. 2021; Doriya et al. 2022). Fruit‐derived fibers play a major role in the modulation of gut microbiota because greater microbial diversity has been correlated with better gut health and resistance to disease.

## 6 Role of Fruit‐Based Diet: Gut Inflammation

**FIGURE 3:**
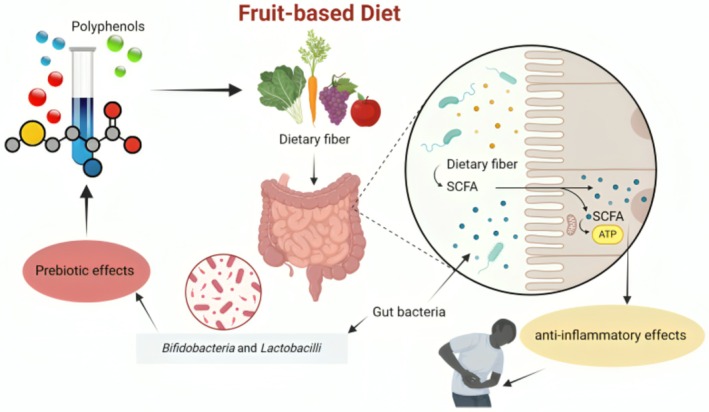
Fruit‐based diet effects on gut inflammation.

A diet rich in fruits has been demonstrated to reduce gut inflammation and symptoms of gastrointestinal disorders, such as IBD. In many cases, an imbalance between good and bad gut microbiota, called dysbiosis, causes inflammation, leading to immunological dysregulation and overproduction of pro‐inflammatory cytokines (Alami et al. 2022). Such effects can be mitigated by consuming fruits possessing strong anti‐inflammatory and antioxidant properties, including reducing oxidative stress and restoring microbial balance (Figure 3) (Liu 2013). By inhibiting NF‐κB, a pathway vital for producing inflammatory mediators such as TNF‐α and ILs, it has been shown that polyphenols, bioactive compounds found within fruits such as berries, grapes, and apples, inhibited gut inflammation by blocking NF‐κB signaling (Rodríguez et al. 2021). They are also substrates for microbial fermentation, which gives rise to bioactive compounds that can regulate immune reactions and strengthen the gut barrier (Crowe‐White et al. 2016). In IBD models, diets of fruits high in fiber and polyphenols have been reported to suppress inflammation markers such as calprotectin and CRP drastically (Lakshmanan et al. 2022).

A diet rich in fruits reduces gut inflammation by strengthening the intestinal barrier and encouraging a balanced gut flora. Dietary fibers, polyphenols, and other bioactive constituents in fruits work as prebiotics, promoting good gut flora and maintaining immunological homeostasis (Olazcuaga et al. 2023). Citrus fruit polyphenols, for example, have been established to possess anti‐inflammatory properties by enhancing the composition of the gut microbiota and controlling the production of pro‐inflammatory cytokines (Miles and Calder 2021; Pap et al. 2021). Fruit antioxidants such as those in berries work against reactive oxygen species (ROS), thus lowering inflammation by reducing oxidative stress within the stomach (Pap et al. 2021). The fabrication of SCFAs, which reduce gut permeability and avert intestinal inflammation, is also improved by dietary fibers from fruits (Rajoka et al. 2017; Rezende et al. 2021). The bioactive complexes in fruits are joined to enhance the reduction of inflammatory reactions by sustaining a healthy gut environment.

Hesperidin and naringenin, flavonoids in citrus fruits, suppress pro‐inflammatory signals and facilitate the colonization of beneficial flora (Miles and Calder 2021). SCFAs are also anti‐inflammatory and result from the fermentation of fruit fibers by gut bacteria. Butyrate has been shown to suppress HDACs, reducing gene expression that promotes inflammation (Jhee et al. 2019). In addition, fruit‐based diets have been reported to relieve symptoms of diseases other than IBD, such as irritable bowel syndrome (IBS) (Doriya et al. 2022). These benefits are mainly due to their ability to enhance gut motility, decrease intestinal permeability, and reconstitute microbial ecology (Sun‐Waterhouse 2011; Fulton et al. 2016). The high actinidin‐ and fiber‐content kiwi has shown promise in IBS symptom amelioration as it has shown mild laxative properties and has the potential for good microbial health (Hunter et al. 2016). Fermentation of fruit fiber by the gut bacteria leads to the generation of SCFAs critical for both gut and systemic health with specific benefits like immune modulation, anti‐inflammatory effects, and epithelial repair (Dahiya and Nigam 2022).

Fruit‐based diets may help steer clear of inflammatory signaling, restore microbial balance, and decrease oxidative stress in inflammatory illnesses like IBD and IBS (McMacken and Shah 2017; Venter 2023). This, therefore, indicates that having different fruits is essential for the overall health of the gastrointestinal tract because it not only increases the diversity of microbes but also creates a healthy gut environment. Much interest has been recently shown in the nutritional relationship with immune health because fruit‐rich diets could alter immunological responses by enhancing gut health (Sun‐Waterhouse 2011). This review covers some information regarding the effect of fruit‐based diets on gut microbiota, immunological control, and disease prevention. These are focused on by exploring a few fruits and their bioactive compounds toward immune function enhancement.

### 6.1 Immune Modulation via Gut Health

Trillions of bacteria exist within the human gut and have been proven necessary in controlling immune reactions (Sun‐Waterhouse 2011). Other cell types that could potentially be affected by the complexity and composition of the gut microbiome include dendritic cells, T‐cells, and macrophages. This significantly impacts the immune system's development and function (Henning et al. 2017). Essentially, diet influences gut microbiota. Diets rich in fruits, vegetables, and polyphenols are believed to preserve a healthy gut environment. These diets enrich the number of good bacteria that enhance the immune system. It has been proven that some antioxidants and polyphenolic compounds known to affect gut flora positively can be mainly found in fruits, including mangoes, berries, and citrus (Henning et al. 2017; Liu 2013).

A good immunological system depends on gut barrier function, which these substances maintain, having properties that inhibit inflammation (Henning et al. 2017). A fruit‐based diet helps restrain immune responses by creating the right gut microbiome. This increases the body's resistance to infections and ailments affecting the immune system (Liu 2013). Research has shown how consuming fruits high in dietary fiber can influence immune cells, such as T‐cells and macrophages. As a prebiotic, fiber promotes the proliferation of beneficial gut bacteria that produce butyrate and other SCFAs. Lakshmanan et al. (2022) note that these SCFAs also help to regulate T‐cell activity, enhancing immunological tolerance and preventing immunological over‐activation, which can lead to autoimmune diseases (Lakshmanan et al. 2022).

#### 6.1.1 Immunomodulatory Effects

A fruit‐based diet is vital for immunomodulation because it influences the gut microbiota's conformation and function. Vitamins, dietary fibers, and polyphenols in fruits exert as prebiotics that improve the development of good bacteria, thus ornamental to the function of gut‐associated lymphoid tissue (GALT) and immunological modulation overall (Conlon and Bird 2014). Berry fruits have been exposed to affect the gut microbiota, cumulative the level of microbial variety and plummeting the dysbiosis within the gut. This is valuable in chronic inflammatory diseases studied by C. Coutinho‐Wolino et al. (2024). Bioactive elements in these fruits such as flavonoids and anthocyanins have anti‐inflammatory and antioxidant properties and work to keep the intestinal barrier and immunological homeostasis. In addition, diets rich in fruits may affect T‐cell modulation and cytokine production, which would diminish inflammation within the gastrointestinal tract. Some fruits, such as apples and bananas, contain soluble fiber named pectin, which is known to prevent inflammation by its aptitude to control gut flora and overwhelm levels of pro‐inflammatory cytokines present throughout disease circumstances such as IBD (Donadio et al. 2024). SCFAs, which upsurge the function of regulatory T‐cells and improve mucosal immunity are also produced following the consumption of fruit polyphenols (Dahiya and Nigam 2022). Generally, these studies demonstrate how consuming a diet rich in fruit may sustain a well‐balanced flora in the gastrointestinal tract and enhance immune function.

#### 6.1.2 Heat Stress Effect

A fruit‐based diet significantly eliminates the adverse influences of heat stress on gut health by altering the composition of microbiota and intestinal confrontation. In addition, sensitive intestinal permeability, dysbiosis, and systemic inflammation emanate from a disturbance of gut homeostasis produced by heat stress (Das and Nair 2019). Fruits rich in polyphenols, such as apples, berries, and citrus fruits, encourage the expansion of good bacteria and diminish oxidative stress, thereby supporting intestinal reliability (Coutinho‐Wolino et al. 2024). In addition, fruit‐based dietary fiber is a prebiotic that improves microbial assortment and gut barrier function two—serious aspects of averting heat‐induced gastrointestinal disorders (Conlon and Bird 2014). Moreover, antioxidants obtained from fruits are flavonoids and carotenoids, which have anti‐inflammatory properties that tend to regulate the immune system of organisms in the presence of high temperatures (Dreher 2018). Generating more ROS in the stomach has been associated with causing inflammation and inadequate nutrient absorption, particularly in persons under heat stress (Dahiya and Nigam 2022). Consumption of fruit‐based diets supplemented with these bioactive compounds has been shown to preserve the balance of gut microbiota and neutralize oxidative stress. Furthermore, whole fruits balance out electrolyte disturbances and fluid loss caused by heat stress by confirming the absorption of essential vitamins and moisture. In short, this demonstrates how fruit‐based nutrition can act as a dietary approach to promote intestinal health under hot situations.

### 6.2 Gut‐Immune Pathways: Disease Prevention

Evidence has been growing that dietary fruit intake may protect against various immune‐related conditions: infections, autoimmune diseases, and allergies. Berries, mangoes, citrus, and other fruits and their consumption have been identified by research as having effects in reducing inflammation, thereby possibly alleviating chronic conditions such as type 2 diabetes and heart disease (Satija and Hu 2018; McMacken and Shah 2017). Their bioactive ingredients may modify the immune systems, reduce oxidative stress, and reduce inflammation—all factors of major importance in developing many chronic diseases. For example, citrus fruits contain flavonoids and vitamin C, which have been proven to boost the immune system to fight against diseases, particularly respiratory infections (Miles and Calder 2021). Since mangoes are also a good source of vitamin A and carotenoids, they enhance mucosal immunity and protect the integrity of the gut lining, thus being significant in preventing infections (Maldonado‐Celis et al. 2019).

Fruit‐based diets have been proven to hold promise in reducing inflammatory markers and alleviating symptoms of autoimmune diseases whereby the immune system mistakenly attacks its cells. The study shows that polyphenolic compounds in fruits such as grapes and apples prevent pro‐inflammatory pathways from being activated, preventing flare‐ups of autoimmune attacks (Liang et al. 2023; Henning et al. 2017). Moreover, the high fiber content of fruit‐based diets promotes the synthesis of SCFAs, which are important for controlling immunological tolerance and stopping the body's tissues from being attacked by the immune system (Sireswar et al. 2021). It has been shown that fruit‐based diets help individuals suffering from allergies, especially concerning environmental and food allergens. The immune system's hypersensitivity toward allergens might be modulated by the anti‐inflammatory properties of fruits, which delay allergic reactions (Venter 2023). A fruit‐based diet can improve gut health and the growth of good bacteria and help fortify the defense of the gut, reducing allergic reactions and systemic inflammation.

### 6.3 Antioxidant‐Rich Fruits and Immune Function

Antioxidants, including vitamin C, flavonoids, and carotenoids in fruits, help enhance immunity and protect the human body from oxidative damage (Miles and Calder 2021). For the immune system to function, defensive systems and oxidative stress must be well balanced. Immune cells function to neutralize and eliminate dangerous chemicals from the body when presented with infections or stressors (Henning et al. 2017). If left unchecked, this could produce free radicals and ROS, resulting in inflammation, cellular death, and immunological dysfunction. The substance, therefore, reduces oxidative stress by neutralizing free radicals (Venter 2023). Antioxidants are protective substances that enhance immune cell activity and protect bodily tissues from damage. Vitamin C, one of the most prominent antioxidants, strengthens the immune system (Liu et al. 2025). The vitamin helps immune cells perform their proper functions, such as neutrophils, lymphocytes, and macrophages. This vitamin facilitates cytokine production and makes it easier for the immune cells to activate and multiply during infection (Mishra et al. 2022).

All of this aside, vitamin C enhances the body's ability to fight oxidative stress by supporting the regeneration of other antioxidants. Citrus fruits, kiwis, and berries are good vitamin C sources (Miles and Calder 2021). The immune system is also backed by flavonoids, another group of antioxidants found in fruits. The immune system is supported by these bioactive substances' anti‐inflammatory and antioxidant properties, which include quercetin, kaempferol, and anthocyanins. The immunological response is regulated through the modulation of the function of immune cells, such as dendritic cells and macrophages, which are fundamental for identifying and reacting to infections, by flavonoids (Miles and Calder 2021). In addition, flavonoids have been shown to enhance the immune system's protection against disease by improving the integrity of the gut barrier, which excludes pathogens from the circulation and halts systemic inflammation (Zheng and Wang 2021). Flavonoids are common in berries, grapes, and apples. Carotenoids are antioxidants that include lutein, zeaxanthin, and beta‐carotene. They play a role in maintaining the integrity of mucosal surfaces, including those found in the gastrointestinal and respiratory systems. These are the body's first lines of defense against infection, and the body converts these substances into vitamin A. Beta‐carotene, among others, has been proven to enhance the body's ability to produce immune cells and then fight off pathogens (Sherwin et al. 2018). Carotenoid‐rich foods, including carrots, papayas, and mangoes, bring immune‐boosting benefits. Therefore, antioxidants in fruits play a crucial role in the defense of immune cells against oxidative damage and strengthen the body's immunity against diseases and infections. Intake of fruits rich in antioxidants will help people improve their immunity and reduce the risk of immunological‐related diseases.

### 6.4 Fruit‐Derived Prebiotics and Immune Health

In recent years, there has been a surge in interest in the role of gut microbiota in maintaining immunological integrity (Sanz et al. 2015). Trillions of bacteria constitute the gut microbiome, crucial for modulating immune responses and maintaining balance in the gut (von Martels et al. 2017). It acts as an immunoprotector through the inhibition of pathogenic microorganisms and stimulates the development of immune cells by regulating cytokine synthesis (Afzaal et al. 2022). Fruits contain prebiotic fibers that maintain gut flora balance and create a friendly environment for immune activity. Prebiotics are non‐digestible food fibers in fruits and other foods that promote the growth and activity of beneficial gut microbiota. These include FOS, pectin, and inulin (Venter 2023). They feed probiotics, beneficial bacteria that live in the gut. When ingested, prebiotics selectively enhance the proliferation of beneficial bacteria such as Lactobacilli and *Bifidobacteria* and inhibit the proliferation of pathogenic microorganisms (von Martels et al. 2017). Thus, the normal functioning of the immune system requires a healthy gut microbiota supported by the selective promotion of beneficial bacteria. One of the many ways the gut‐immune system interacts is through SCFAs, which are produced when gut bacteria ferment prebiotics. SCFAs, such as butyrate, acetate, and propionate, are reported by Sherwin et al. (2018) to possess anti‐inflammatory properties that contribute to preserving the intestinal barrier function, preventing harmful components from being transferred to the blood (Sherwin et al. 2018). Fruit‐based prebiotics enhance the synthesis of such SCFAs through modulation of gut flora, improved immunity, and reduced inflammation. Besides promoting beneficial flora, prebiotics influence the immune system by impacting GALT, an essential element of the body's immunological defense framework. Prebiotics help strengthen the body's immunity to diseases (Zhang et al. 2018).

GALT regulates antibody production and immunizations (Vancamelbeke and Vermeire 2017). For example, it has been shown that supplementation with fruit prebiotics increases the activity of immune cells such as T‐lymphocytes and macrophages, which help detect and remove infections (Sanz et al. 2015). Fruits like citrus fruits, apples, and bananas contain high prebiotic fibers. For instance, apples are rich in soluble fiber pectin, which acts as a prebiotic, aiding the growth of good gut flora (Zhang et al. 2021). Green bananas are a good source of prebiotics due to the resistant starch that they feed good intestinal bacteria. Citrus fruits have several prebiotic and antioxidant effects that enhance immune system function due to their vitamin C and prebiotic fiber content.

Since stress has been shown to influence gut microbiota and the function of the GI tract, it is thus important for gut health. Among gastrointestinal problems caused by stress, particularly long‐term psychological stress, are IBS, IBD, and other functional gastrointestinal diseases. According to Yoo et al. (2020), the stress reaction points to the discharge of cortisol and other stress hormones, which can change intestinal permeability, gut bacteria balance, and gut motility (Yoo et al. 2020). According to Vancamelbeke and Vermeire (2017), the stress‐induced variations in the gut microbiota conformation can upsurge the proliferation of pathogenic bacteria and decrease the population of helpful bacteria. It results in inflammation, augmented gut permeability, and a broken gut barrier (Vancamelbeke and Vermeire 2017). Moreover, psychological stress can influence gut function due to the existence of the vagus nerve and other neurological routes because of the gut–brain axis that connects the gut to the CNS (Sherwin et al. 2018). Because this is a mutually influencing relationship, stress can thus have a worse impact on gut health. In contrast, gut dysfunction would increase the feeling of stress and anxiety, setting up a vicious cycle (Yoo et al. 2020). Stress management is essential for maintaining gut health (Bandelow and Michaelis 2015).

Several interventions, including dietary changes, mindfulness practices, and lifestyle changes, have been shown to reduce stress levels and improve gut health effectively. For instance, dietary methods involving probiotics, prebiotics, and appropriate high‐fiber foods can modify the gut microbiota and enhance the gut barrier (Rajoka et al. 2017). Mindfulness‐based activities, such as yoga and meditation, have also decreased cortisol levels, improved gut health, and reduced symptoms of IBS and other gastrointestinal diseases caused by stress (Chong et al. 2024). Exercise is another excellent stress reduction technique as it has been linked to reduced inflammation and augmented diversity of gut microbiota, improving gut health and overall well‐being (Tapsell et al. 2016). Lastly, it has been proven that social support and cognitive behavioral therapy can reduce stress‐related psychological and harmful gut‐brain axis effects (Bandelow and Michaelis 2015). As such, people can significantly improve their gut health, ability to resist the adverse effects of stress, and general health outcomes with these techniques applied in daily life. Stress impacts intestinal permeability, inflammation, and gut microbiota, crucial for gut health. However, with engrossing therapies like dietary variations, mindfulness, exercise, and psychological provision, the negative influence of stress on gut health can be concentrated. These techniques promote healthy gut flora, overall health, and emotional stability.

To summarize, fruit‐derived prebiotics are essential in modifying gut microbiota and enhancing the immune system and stress levels. Such prebiotic fibers provide the basis for immune system strength and resilience by improving gut barrier function, promoting beneficial bacteria, regulating stress levels, and regulating immunological responses (Zhang et al. 2018). Therefore, dietary inclusion of fruits rich in prebiotics can be a valuable strategy to maintain the integrity of the immune system. This process indicates IBD to involve chronic inflammation of the gastrointestinal system. IBD is a chronic disorder whose etiology encompasses immunological, environmental, genetic, and microbiota‐related factors, including Crohn's disease and ulcerative colitis. Dietary therapies in managing IBD and stress have recently gained greater attention, focusing on reducing inflammation and enhancing gut health. Of all these treatments, fruit‐based diets have been investigated, considering the potential benefits of their composition, which contains fiber, antioxidants, and anti‐inflammatory properties. As such, this review gathers as much information as possible that has been documented on the treatment of IBD using fruit‐based diets. Since diet plays a central role in the management of IBD, considering how some foods can either be pro‐inflammatory or anti‐inflammatory. Hence, an anti‐inflammatory diet is necessary to control the inflammatory response and flare‐ups. The gut microbiota plays a crucial role in the onset and course of IBD, and diet‐related factors such as fiber, fats, and antioxidants can modulate it (Zhang et al. 2018). Dietary interventions that either directly modify immune responses or foster a healthy gut microbiota may influence the natural history of IBD (O'Mahony et al. 2020).

## 7 Fruits: Prominent Effects on Gut Health and Immunity

### 7.1 Berries (Blueberries, Strawberries, Raspberries)

Berries, such as strawberries, blueberries, and raspberries, are valued for their contents of antioxidants, polyphenols, and dietary fibers (Lavefve et al. 2020). Research has shown that such compounds are crucial in regulating the gut microbiota and are vital for maintaining digestive and immune systems (Satija and Hu 2018). Berries contain such polyphenols as flavonoids and anthocyanins with robust antioxidant capabilities. As oxidative stress is reduced, these agents promote gut health and inhibit inflammation (Afzaal et al. 2022). Polyphenols increase the proliferation of beneficial bacteria and create a wholesome gut environment that enhances immune function by modulating the gut microbiota (Pap et al. 2021). Inflammation pathways are also regulated by the bioactive compounds in berries for immune health (Govers et al. 2018). This research has proved that berries influence gut microbiota composition by developing beneficial bacteria such as *Lactobacillus* and *Bifidobacterium* spp. Gut integrity and increased synthesis of SCFAs, which contribute to immune function, remain dependent on this microbiota modulation (Chen et al. 2022). More of these helpful microbes can help maintain the balance of the gut microbiome and even prevent harmful pathogens from growing (Lavefve et al. 2020). Fiber, which is found in berries, regulates the immune system.

When the gut bacteria ferment fiber, SCFAs with anti‐inflammatory qualities are produced. These SCFAs enhance the function of regulatory T‐cells, which are critical for maintaining immunological homeostasis and preventing over‐exuberant inflammatory responses (Komarnytsky et al. 2023). Moreover, berry antioxidant properties have recently been shown to affect immune cells directly, enhancing their function in fighting infections and decreasing inflammation (Bouyahya et al. 2022). Based on a review of randomized controlled trials by Marino et al. (2024), administering blueberries to healthy adults significantly ameliorated their gut microbial profile and reduced inflammatory markers (Marino et al. 2024). In their 2024 study, Coutinho‐Wolino et al. (2024) examined the impact of fruits, such as blueberries and raspberries, on gut dysbiosis in patients receiving treatment for chronic renal disease (Coutinho‐Wolino et al. 2024). The results indicated that berry consumption enhances gut microbiota, associated with enhanced immunological responses and reduced inflammation. Such findings suggest that berry consumption can significantly improve gut health and the immune system (Coutinho‐Wolino et al. 2024).

### 7.2 Citrus Fruits (Oranges, Lemons, Grapefruit)

Oranges, lemons, grapefruits, and other citrus fruits are rich in flavonoids, terpenes, limonoids, and vitamin C (Lu et al. 2023). Due to these chemicals, citrus fruits are an essential dietary constituent for gut health and immunological functions and greatly contribute to their anti‐inflammatory and immune‐enhancing properties (Barreca et al. 2020). Citrus fruits contain vitamin C in the right amount, which may contribute to having a tremendous immunopotentiating antioxidant system. In addition, the above aspects enhance immune cell activities and improve skin strength and permeability, as this may limit access to germs. Its antioxidant quality has also reduced stress induced by oxidative elements since immunologic functioning would be undermined otherwise when these stress‐causing substances are oxidatively in use (Miles and Calder 2021). General immunity is supported by regular intake of citrus fruits as inflammation decreases and immunological responses become regulated (Sanofer 2014).

The anti‐inflammatory properties of flavonoids such as quercetin and hesperidin, rich in citrus fruits, have been shown to act in the gut (Miles and Calder 2021). By inhibiting pro‐inflammatory cytokines and maintaining the integrity of the gut barrier, these bioactive compounds help attenuate intestinal inflammation. Citrus fruit flavonoids decrease intestinal permeability and change the gut microbiota, thus reducing IBDs and other digestive disorders (Barreca et al. 2020). The health benefits of citrus fruits are due to limonoids, terpenes, and carotenoids along with vitamin C. Powerful antioxidants, especially the beta‐carotene carotenoids, boost immune activation and decrease oxidative stress and intestinal inflammation. Citrus peels contain various terpenes, including limonene, and have been shown to enhance the body's defense mechanisms against infection (Patil et al. 2017). These compounds enhance gut health by encouraging beneficial bacteria growth and increasing SCFAs, essential for immunological activities (Lu et al. 2023).

As Saini et al. (2022) reported, citrus fruits can modify intestinal health. Flavonoids, carotenoids, and other citrus bioactives reduced intestinal inflammation and oxidative stress indicators, promoting healthy gut flora (Saini et al. 2022). Bellavite and Donzelli (2020) studied the immunoprotective properties of citrus fruits, focusing specifically on their role in enhancing immunological responses to respiratory pathogens. The study may indicate that the citrus flavonoid hesperidin could also exert some anti‐inflammatory effects and inhibit the replication of SARS‐CoV‐2 viruses (Bellavite and Donzelli 2020). According to a systematic review by Maugeri et al. (2019), grapefruit consumption enhances immune function, particularly in preventing oxidative stress and reducing inflammation markers associated with aging or inflammation (Maugeri et al. 2019).

The synergistic effect of flavonoids, carotenoids, terpenes, and vitamin C is mainly attributed to the fact that citrus fruits can improve the function of the immune system and gut health along with reducing inflammation and increasing resistance to infection (Maugeri et al. 2019). Studies have shown these compounds to have antioxidant and anti‐inflammatory properties that, in a straight line, provide the immune system and gut (Patil et al. 2017). Specifically, flavonoids, including quercetin and hesperidin, abundant in citrus fruits, have been found to inhibit pro‐inflammatory cytokines and retain gut barrier integrity, thus reducing intestinal absorptivity (Barreca et al. 2020). These substances reduce the intestines' permeability to stop harmful pathogens from entering the body and reduce chronic inflammation, a significant contributor to digestive issues and IBD (Lu et al. 2023). In addition, vitamin C and the carotenoids in citrus fruits, particularly beta‐carotene, assist as potent antioxidants, which decrease oxidative stress, which would damage gut cells and exacerbate inflammation (Patil et al. 2017). By cumulative immunological activation, these antioxidants endorse the function of the gut as a vital immune organ and assist in fighting infections (Maugeri et al. 2019). Citrus fruit compounds, including terpenes such as limonene in citrus peels, have also been shown to enhance the body's defense mechanisms against pathogens by boosting the development of good gut bacteria and cumulative the production of SCFAs, which are vital for immune system modulation (Lu et al. 2023). Citrus fruits can also alter the gut microbiota composition to a more balanced state associated with lower inflammation (Saini et al. 2022). Citrus fruits promote gut microbiota by enhancing the growth of beneficial bacteria, which is vital for systemic lowering of inflammation and the boosting of immunological responses. Maugeri et al. (2019) have demonstrated that grapefruit consumption has protective effects on immunological function by reducing oxidative stress and inflammatory markers, which are often elevated in aging (Maugeri et al. 2019). This “inflammaging” reduction is essential to enhance the body's confrontation with infections and chronic diseases. Citrus fruits endorse gastrointestinal health, establish the immune system, and decrease systemic inflammation, and they have a multimodal defensive impact that raises resistance to infections.

### 7.3 Apple and Pear

Pectin is a soluble fiber found in apples and pears, essential for controlling the immune system and the health of the gastrointestinal tract. Pectin, which is rich in the skins of these fruits, has attracted attention because it may enhance immune function and alter the composition of gut flora (Koutsos et al. 2015). A soluble fiber called pectin that ferments in the colon encourages two types of good gut bacteria: *Lactobacillus* and *Bifidobacterium*. It contributes to preserving intestinal integrity and lowering inflammation through SCFAs, such as butyrate, as byproducts of this fermentation. Studies have shown that consuming fruits containing pectin, including apples and pears, may improve intestinal barrier function and alleviate symptoms of IBS (Blanco‐Pérez et al. 2021).

Additionally, pectin has been related to stabilizing the gut microbiota, an action required to maintain a healthy immune system (Beukema et al. 2020). The habitual consumption of apples and pears is related to alterations in gut microbiota. Rich in pectin, diets are supportive of good bacteria growth, repressing aggressive pathogens to help in the maintenance of a healthy microbiome. A healthy microbiome is fundamental in the modulation of immunity because it directly impacts the formation of immune cells and the control of inflammation (Koutsos et al. 2015). In addition, it can stimulate the immune system as a prebiotic by promoting the growth of some beneficial bacteria that regulate the immune system (Sauceda et al. 2017). The influence exerted by pectin on gut microbiota remains its primary way to augment the immune system. SCFAs, produced due to pectin fermentation, help activate regulatory T‐cells. Such cells are vital in avoiding overreactive immune responses and chronic inflammation (Beukema et al. 2020). In addition, pectin and its oligosaccharides have been shown to directly affect the immune cells by making them better at combating infections and decreasing the inflammation caused by these pathogens. This can be especially useful in allergies and inflammatory conditions (Elshahed et al. 2021; Beukema et al. 2020).

Moreover, studies suggest pectin may possess anti‐inflammatory activities vital to maintaining immunological equilibrium in vulnerable populations, such as newborns and preterm infants (Donadio et al. 2024). According to Dreher (2018) study on the effects of whole fruit and fiber on gut health, pectin from apples and pears promotes gut integrity and microbial diversity. This, in turn, helps maintain conditions such as IBS and digestion in general. According to other studies, consuming apple pectin has been shown to alter gut microbiota by increasing the production of SCFAs and modifying inflammatory markers (Blanco‐Pérez et al. 2021). According to this research, dietary therapies may aid persons with diminished immune responses by improving their responses. Hikawczuk et al. (2024) assert that pectin oligosaccharides from fruits such as apples and pears stabilize gut microbiota and minimize inflammation, which benefits the immune system, especially in inflammatory diseases. These findings highlight several benefits of consuming apples and pears, especially enhancing immunity, modulating microbiota composition, and maintaining gut health.

### 7.4 Banana

FOS and resistant starch are rich components of bananas, especially when green or ripe. These contribute to immune support and gut microflora modulation. These two prebiotics encourage the multiplication and activity of beneficial gut flora (Chong et al. 2024). They help keep immunity intact. The RS of bananas, the starch that stays undigested in the small intestine, reaches the large intestine. It is one of the SCFAs produced from the gut microbiota's fermentation of RS, which is already in the colon. Butyrate decreases inflammation, maintains intestinal motility, and preserves the integrity of the gut (de Andra et al. 2021).

Furthermore, RS feeds beneficial bacteria such as Lactobacilli and *Bifidobacteria*, enhancing microbiota diversity and promoting healthy gut flora (Chong et al. 2024). FOS, a prebiotic fiber found in bananas, particularly encourages the growth of beneficial gut bacteria, *bifidobacteria*. These bacteria are essential for immune system regulation because they enhance mucosal immunity and produce SCFAs. It has been shown that FOS enhances metabolic well‐being and satiety by increasing the production of gut‐derived hormones such as GLP‐1 (Assudani 2019). Moreover, low levels of gut‐derived uremic toxins were observed during the consumption of FOS, which helped reduce the chances of inflammation and immunological dysregulation (Xiong et al. 2022; de Andra et al. 2021). The diverse and balanced gut flora, supported by the prebiotic activity of RS and FOS, is accountable for healthy immune function. This healthy microbiome controls the immunological responses by generating anti‐inflammatory SCFAs and modulating immune cell activities, including regulatory T‐cells (Assudani 2019). Research studies show that RS and FOS promote gut health and enhance the gut's immune surveillance function through mucosal immunity production that protects the body from infections and chronic diseases (Rezende et al. 2021).

To determine how unripe banana flour affects gut‐derived uremic toxins in patients undergoing peritoneal dialysis, de Andra et al. (2021) conducted a randomized, double‐blinded study. To this end, unripe banana flour rich in resistant starch can enhance immunities and gut health by reducing toxic matter originating in the gut and altering the microbiota (Fan et al. 2024). In addition, FOS‐supplemented, well‐known Indian meals have enhanced immune function, augmented satiety hormone levels, and altered gut microbiota in young obese men by favoring beneficial microflora (Assudani 2019). This showed how FOS could enhance gut health and immunity. Uraipan et al. (2014) investigated the synergistic effects of *Lactobacillus plantarum* and green banana starch in the proximal colon in 2014. The study found that resistant starch in green banana starch enhanced the probiotic activity of *Lactobacillus plantarum* , enhancing gut health and protecting against harmful bacteria such as *Salmonella Typhimurium* . This shows how green bananas can enhance immunity and support a healthy microbiome. Yuen (2021) explored the survival and growth of gut‐dwelling probiotic bacteria using inulin, FOS, and other fibers. It was also observed that FOS extracted from bananas greatly promoted the survival and proliferation of good probiotic yeast and bacteria, thus supporting its role in promoting gut health and modifying immunological activities. A similar study by Chong et al. (2024) has reported that consuming green banana and pineapple fiber powder enhanced the host's gut flora, further supporting the merits of banana fibers, specifically their prebiotic potential.

### 7.5 Pomegranate

Pomegranates ( *Punica granatum* ) are rich in polyphenols such as flavonoids, tannins, and anthocyanins. It has been shown that pomegranates have anti‐inflammatory and antioxidant properties. Bioactive compounds greatly influence the decrease in gut inflammation, immune system modulation, and overall health enhancement (Wu et al. 2019). Due to their potent effects on microbiota and the ability to counter oxidative stress, pomegranates can influence the immune system and gut health. Pomegranate polyphenols have been significantly researched for their ability to lower oxidative stress and intestinal inflammation. These compounds can alter the gut microbiota, promoting the proliferation of positive bacteria. Studies show that pomegranate polyphenols can inhibit systemic inflammation and gastrointestinal diseases by decreasing pro‐inflammatory cytokines and maintaining the integrity of the intestinal lining (Santacroce et al. 2024).

In addition, the antioxidant property of pomegranate polyphenols facilitates reducing intestinal inflammation, which is vital in maintaining an intact immune system (Yu et al. 2024). It has been shown that pomegranate polyphenols, especially those in the peel, exert immune‐enhancing effects through the immunomodulation of cells like T‐cells, B‐cells, and macrophages (Santacroce et al. 2024). The body utilizes this effect to enhance its immune capacity against chronic diseases and infections. The anti‐inflammatory effect of pomegranates also enhances the modulation of the immune system, preventing immunological dysregulation that can cause autoimmune diseases or chronic inflammation (Wu et al. 2019). Wu et al. (2019) investigated pomegranate peel polysaccharides' immunomodulatory and antioxidant properties in immunosuppressed mice. The polysaccharides had potent antioxidant activity and significantly enhanced immunological responses, restoring the immune system in immunocompromised mice. Such findings give credence to using pomegranate peels as an immunoenhancing medication. Yu et al. (2024) investigated the effects of fermented pomegranate peel polyphenols on white shrimp, *Litopenaeus vannamei* , and reported improved growth performance, immunological responses, and disease resistance in the shrimp. With its implications for applying pomegranate polyphenols to enhance immunological function in humans and animals, this study provides further opportunities for pomegranate extracts (Yu et al. 2024).

In 2022, AKURU considered examining the influence of pomegranate peel powder on broiler chickens' growth performance, gut integrity, and immune response. The author concluded that the component of pomegranate improves gut inflammation and immune support as it improves markers of general health, immune efficiency, and gut health of hens. Besides, pomegranate polyphenols have been found to benefit intestinal microbiota. Pomegranate polyphenols were demonstrated to increase the number of beneficial bacteria, such as *Bifidobacteria* and Lactobacilli, necessary for regulating inflammatory and immunological responses. A healthy gut microbiota maintains immune homeostasis, and pomegranate polyphenols contribute to this balance by supporting a diverse and stable microbial population (Singh et al. 2023). The antioxidant properties of pomegranate polyphenols also influence the regulation of the immune system. Pomegranates scavenge free radicals and reduce oxidative stress, which protects cells from damage and maintains the integrity of the intestinal lining. This reaction is vital for immune function since exaggerated oxidative stress is believed to lead to inflammation, immunological failure, and increased susceptibility to infections (Soory 2009).

### 7.6 Papaya and Pineapple

The two tropical fruits, papaya ( *Carica papaya* ) and pineapple ( *Ananas comosus* ) harbor digestive enzymes in the form of proteolytic papain and bromelain. Proteolytic enzymes are essential for digestion, gut health, and immunological support because they break proteins into smaller peptides and amino acids, allowing nutrient absorption and reducing inflammation (Giangrieco et al. 2023). Additionally, they support the integrity of the gut barrier due to their anti‐inflammatory properties, thereby preventing leaky gut and maintaining gastrointestinal health. Other than digestive benefits, papain contains immunomodulatory properties. Studies indicate that papain may enhance immunological responses by regulating immune cells such as macrophages and lymphocytes. Papain has also been established to reduce inflammation and promote healing in many inflammatory conditions (Silva‐ López and Gonçalves 2019). Bromelain has been found to have anti‐inflammatory and immune‐stimulating effects. It is also acknowledged to increase the production of cytokines and the activity of white blood cells, which protect the body from infections.

Furthermore, since it has been shown in some studies to reduce systemic inflammation, patients with chronic inflammatory diseases have been using it (Olivier 2015). Giangrieco et al. (2023) explored the responses of human immune systems to cysteine proteases derived from papain found in papaya and pineapple. The study claims that pineapple's bromelain has a substantial anti‐inflammatory property, which reduces allergy reactions and maintains a strong immune system. This study's results support the view that enzymes derived from pineapple can enhance immunity and reduce inflammation. Papain and bromelain are other proteolytic enzymes considered for reducing pain and inflammation. As stated by Olivier (2015) report, bromelain and papain have been used as medical treatments for diseases like osteoarthritis and rheumatoid arthritis due to their anti‐inflammatory properties and being an alternate natural NSAID. In the clinical study by Sarrimanolis 2023, bromelain can be utilized as an additive in poultry feeds; this improves the health status and nutrient digestibility of broiler chickens (Sarrimanolis 2023). Therefore, this work represents the extensive potential of bromelain in improving digestion in animals and health conditions, which, in turn, affects humans. In addition to digestion, papain and bromelain have been used medicinally. They treat diseases such as wounds, chronic inflammation, and some cancers (Conlon and Bird 2014). In particular, bromelain has been studied for its potential role in enhancing immunity and alleviating asthma and other allergies (Ahmad et al. 2018). Also, papaya and pineapple enzymes are commonly found in natural supplements designed to enhance immune system strength and improve digestion. Table 2 shows different fruits' impact on immune function.

**TABLE 2:** Impact of fruits on gut health and immune function.

Fruit	Impact	Reference
Berries	Rich in antioxidants, polyphenols, and fiber, berries modulate gut microbiota composition, reduce inflammation, and support immune health	Bohn et al. (2024)
Citrus Fruits (Oranges, Lemons, Grapefruit)	High in Vitamin C and flavonoids, citrus fruits reduce gut inflammation, support immune function, and improve gut barrier integrity	Fazio et al. (2021)
Apples and Pears	Rich in pectin fiber, apples and pears have prebiotic effects, modulate gut microbiota, and enhance immune function	Slavin (2013)
Bananas	Contain resistant starch and fructooligosaccharides (FOS), promoting beneficial gut bacteria, aiding digestion, and supporting immune function	Meyer et al. (2020)
Pomegranates	Rich in polyphenols, pomegranates reduce gut inflammation, enhance gut microbiota diversity, and support immune health	Salehi et al. (2019)
Papaya and Pineapple	Contain digestive enzymes (e.g., papain, bromelain) that support digestion, reduce gut inflammation, and promote gut health and immune function	Ziegleret al. (2021)
Kiwifruit	Rich in fiber, vitamin C, and antioxidants, kiwifruit supports gut health, improves digestion, and enhances immune function through its prebiotic effects	Chen et al. (2022)
Mango	High in vitamin C, polyphenols, and dietary fiber, mangoes have anti‐inflammatory properties, support gut health, and enhance immune responses	Saini et al. (2022)
Grapes	Rich in polyphenols (e.g., resveratrol), grapes support gut microbiota balance and help protect against oxidative stress and inflammation	Zhao et al. (2020)
Avocados	High in healthy fats, fiber, and antioxidants, avocados support gut health by reducing inflammation, improving gut microbiota composition, and promoting gut barrier function	Jakubowicz et al. (2024)
Watermelon	Contains lycopene, an antioxidant that reduces oxidative stress and inflammation, helping improve gut health and immune function	Crupi et al. (2023)
Tomatoes	Rich in lycopene, tomatoes help modulate gut microbiota, reduce gut inflammation, and provide antioxidant protection to support immune function	Krawczyk et al. (2022)

Though the consumption of fruits contributes to gut health, there are other factors. A well‐balanced diet, which provides sufficient dietary fiber, probiotics, and prebiotics, helps keep gut microbiota healthy (Ahmad et al. 2018). For example, dietary fiber increases microbial variety as it serves as a food source for beneficial gut bacteria. Through feeding the good bacteria, a high‐fiber diet rich in fruits, vegetables, and whole grains has been seen to improve the function of the gut and reduce gastrointestinal disorders (Conlon and Bird 2014). Additionally, some other foods of great importance are probiotic‐enriched with high contents, including kefir, yogurt, and fermented vegetables (Bäckhed et al. 2012). Beneficial live bacteria found in these foods help the body restore the gut's microbial equilibrium, particularly after undergoing antibiotic therapy and during periods of irregular digestion. As Alonso and Guarner (2013), they help boost intestinal barrier permeability, decrease inflammatory markers, and increase immune cell response (Alonso and Guarner 2013).

Additionally, consuming prebiotics undigested dietary ingredients in foods such as bananas, garlic, and onions nourishes beneficial gut bacteria and enhances gut microbiota health (Aziz et al. 2013). Fats and proteins should also be used in the diet to support gut health, alongside fiber and probiotics (Bäckhed et al. 2012). Intake of omega‐3 fatty acids has been linked to decreased inflammation and improved intestinal barrier function with increased consumption, found in walnuts, flaxseeds, and fatty fish (Bäckhed et al. 2012). On the other hand, a well‐balanced intake of protein from plant‐based sources such as legumes and nuts can support a diverse gut microbiome. In contrast, extreme intake of animal fats can destructively impact gut health by promoting the growth of harmful bacteria (Fouhse et al. 2016). Lastly, gut health maintenance includes steering clear of processed meals and sugars. Consuming large amounts of these items may lead to an imbalance in gut microbiota, known as dysbiosis, related to many gastrointestinal and systemic conditions, such as obesity and IBD. This balance in gut function and overall health may be maintained by eating less of these items and replacing them with whole, nutrient‐dense foods that can enhance the growth of a healthy microbiome (Koutsos et al. 2015).

Some fruit wastes and peels contain bioactive compounds that, while not part of the fruit itself, have potential benefits to intestinal health. An example is the banana peel, which is rich in antioxidants, polyphenols, and fiber. Scientific research indicates that it is the relatively high fiber content, especially in banana peels, that acts as a prebiotic, thus promoting the development of *Lactobacilli* and *Bifidobacteria*, which are two beneficial gut microbiota (Li et al. 2025; Sivakanthan et al. 2022). Moreover, polyphenols in the banana peels have anti‐inflammatory properties that may alleviate IBD and IBS patients by reducing gastrointestinal inflammation (Sivakanthan et al. 2022). Furthermore, the peels may possess antioxidant properties that decrease oxidative stress; therefore, there is an augmented chance of supporting the immune system and gastrointestinal well‐being (Patil et al. 2017). The peels and seeds of citrus fruits are also known to have health benefits for the intestinal system. Hesperidin and polymethoxylated flavones are some of the flavonoids in citrus peels that possess antibacterial, anti‐inflammatory, and antioxidant properties. These compounds may play a role in enhanced digestive health through reduced inflammation and a better balance of gut microbiota (Barreca et al. 2020). An additional fruit leftover that has been researched is pomegranate peel, which contains a high amount of polyphenols. This has shown promise in reducing inflammation, promoting the growth of good bacteria, and adapting gut microbiota (Maugeri et al. 2019). Together, soluble and insoluble fibers in fruit seeds, like those from apples, support the gut's health by forming healthy SCFAs and various microbiota (Lu et al. 2023). Besides helping decrease food waste, these residues contain a wealth of bioactive compounds that may improve the diversity and overall health of the gut.

## 8 Evidence From Clinical and Epidemiological Studies

Over the years, the effects of fruit‐based diets on the immune system and gut microbiota composition have been an issue of enormous concern. The human gut microbiome is a complex mixture of microorganisms required for many physiological functions, such as metabolism, immunity, and digestion. Recent observational studies and clinical trials have shown that a fruit‐rich diet alters the gut flora and improves the immune system. According to such research, a diet rich in fruits can strengthen immune responses and preserve the balance of gut flora. It may result in desirable health effects. Henning et al. (2017) have analyzed the health effects of diets high in fruits and vegetables and their impact on the microbiota. The researchers concluded that the beneficial bacteria, such as *Lactobacillus* and *Bifidobacterium*, would increase significantly by altering the gut microbiota. It is known that these microbes enhance gut health by producing SCFAs, which are helpful for immune regulation and intestinal function. The study revealed that fruit and vegetable juices can alter microbial populations to support digestive and immunological health (Henning et al. 2017). Various clinical studies have found diets rich in fruits improve immune function. For example, Sireswar et al. (2021) found that fruit‐based beverages improved the probiotic *Lacticaseibacillus rhamnosus* GG's ability to reduce DSS‐induced intestinal inflammation in animal models. The study showed how a diet based on fruits, particularly fermentation, may enhance gut conditions, promoting the growth of beneficial probiotics, which may change the level of immune responses (Sireswar et al. 2021).

Olazcuaga et al. (2023) assayed the metabolic impacts of different fruit‐based diets using an insect species in its ability to understand the effects of immunity and metabolism. Given that both the quality and quantity of fruits altered immunological markers along with metabolic markers such as glucose, they highlighted the role played by fruits in immune modulation. Even though conducted on insects, this research provides a general insight into how fruit‐based diets can impact human metabolism and immunity (Olazcuaga et al. 2023). In a cohort study conducted in 2019, Jhee et al. investigated the relationship between human kidney function and a diet rich in fruits and vegetables, focusing on gut bacteria diversity. According to the authors, the people who consumed more fruits and vegetables had a more diverse gut microbiota associated with better health outcomes. A diverse gut microbiome is also known to enhance immune function by promoting microbial populations that help reduce inflammation and prevent infections (Jhee et al. 2019). Table 3 depicts the case studies that link fruit consumption with changes in gut microbiota and immune biomarkers.

**TABLE 3:** Case studies that link fruit consumption with changes in gut microbiota and immune biomarkers.

Study	Subject	Fruit intervention	Outcome	Finding
Ajiboye et al. (2017)	Diabetic rats	*Artocarpus altilis* (breadfruit)	Blood glucose, lipid profile	Reduced blood glucose and lipids, improved gut health markers
Akuru (2022)	Broiler chickens	Pomegranate peel powder	Growth performance, gut integrity, hematological indices	Improved gut integrity and immune function in poultry
Alami et al. (2022)	Patients with NAFLD	Fruit‐rich diet (various fruits)	Liver biomarkers, insulin resistance, lipid profile	Significant improvement in liver function and insulin sensitivity
Alasmar et al. (2019)	Human review	General fruit‐based diet	Gut microbiota diversity	Fruits enhance beneficial gut bacteria diversity, boosting health
Alissa and Ferns (2017)	General population	High fruit and vegetable intake	Cardiovascular risk markers	Improved heart health, associated with positive changes in gut microbiota
Arumugam et al. (2021)	General population	Superfoods (including fruits)	Immune biomarkers, gut microbiota	Fruits enhance immune function, linked with gut microbiota modulation
Beigoli and Boskabady (2024)	General population	Various fruits and vegetables	Immune modulation, inflammation	Natural fruits modulate immune responses and reduce inflammation
Barreca et al. (2020)	Human review	Citrus fruits	Health biomarkers, gut microbiota	Citrus flavones support immune health and modulate gut microbiota
Beukema et al. (2020)	Human study	Pectin from fruits	Immune cells, gut barrier	Pectin promotes gut immunity and microbiota balance
Chen et al. (2022)	General population	Various berries	Antioxidant capacity, gut microbiota	Berry consumption improves antioxidant levels and gut microbiota
Chong et al. (2024)	Healthy adults	Green banana and pineapple fiber	Gut microbiome diversity	Fiber improves gut microbiome diversity and immune markers
Conlon and Bird (2014)	General population	Dietary intervention with fruits	Gut microbiota and immune markers	Fruits enhance gut microbiota and immune function

## 9 Potential Risk of Fruit‐Based Diet in Certain Diseases

Fruits are high in dietary fiber, polyphenols, and antioxidants that favor a healthy microbiome and can offer many benefits for gut health (Zeng et al. 2024; Peluso and Palmery 2014). There are conceivable dangers and limitations to consider when on such a diet. First, excessive intake of fruits results in excessive consumption of sugar, and this may be associated with derangement of gut microbiota; specifically, too much bacteria that ferment sugar for their energy requirements, which will produce gas that may cause bloating and other digestive anxiety (Zang et al. 2023). Moreover, fruits' nutrient composition differs, and the action of their fibers and phytochemicals may also vary regarding gut health (Oliveira et al. 2018). For example, an imbalanced diet comprising fruits may not provide adequate amounts of protein or other essential nutrients, which might cause dietary inadequacies after some time, weakening overall health (Fardet and Richonnet 2020). Second, while fruits have often been associated with positive health effects, certain fruit processing and preservation methods in fruit‐based foods can also be harmful. Fruit‐based drinks, for instance, are commonly pasteurized or undergo some other treatment that could diminish the nutritional content of these beverages, such as their fiber and bioactive compounds loss (Gomes et al. 2021). Moreover, some processed fruit drinks contain artificial sweeteners, preservatives, or extra sugars that reduce the nutritional benefits of the fruit and negatively affect gastrointestinal health (Sireswar et al. 2021). Consequently, even though a fruit‐based diet can advance gut health, it is significant to preserve a varied and balanced diet that deliberates whole fruits and how they are processed to exploit nutritional benefits and diminish risks (Miles and Calder 2021). Although a fruit‐based diet has many health benefits, some individuals with specific medical conditions must be careful or limit their consumption (Gomes et al. 2021). For example, individuals with fructose malabsorption or intolerance may experience gastrointestinal distress, such as bloating, diarrhea, or abdominal pain, when consuming high‐fructose fruits like apples, pears, and some berries (Santacroce et al. 2024). Many often have trouble digesting Fructose, a natural sugar in fruits and fruit products (Sireswar et al. 2021). This leads to an issue of malabsorption; it causes excessive fermentation by colonic bacteria, which worsens complications. Thus, these patients should always limit or keep away from higher‐fructose fruit foods and seek lower‐fructose foods like bananas and citrus fruit (Miles and Calder 2021). Moreover, a fruit‐rich diet should be cautiously implemented by individuals with diabetes or those who control blood sugar levels (Mishra et al. 2022). This is because fruits contain high amounts of natural sugars, such as fructose and glucose, although they are rich in vitamins, antioxidants, and fiber. Consuming high‐glycemic fruits such as grapes or watermelon can cause a short‐term spiking of blood sugar, which will compromise the management of diabetic patients over their blood sugar levels (Santos et al. 2019). Individuals with diabetes should focus on consuming fruits with a low glycemic index. Such fruits are apples and berries. In moderation, they should be part of a generally balanced diet to minimize the risk of swings in blood sugar (Mishra et al. 2022). There is also an issue regarding the consumption of potassium and phosphorus levels, and people affected by renal disease have to refrain from consuming particular fruits. As a result, individuals with chronic kidney disease (CKD) should consider limiting their intake of potassium‐rich foods like bananas, oranges, and avocados to prevent the onset of hyperkalemia (Oliveira et al. 2018). Since the kidneys are incapable of expelling potassium adequately, elevated potassium levels in CKD patients can also be dangerous and cause severe side effects, including cardiac arrhythmias. In such scenarios, healthcare providers must adjust the consumption of fruits according to the patient's renal function and recommend lesser amounts of potassium‐loaded fruits like apples, berries, and grapes. Last but not least, even though fruits are normally considered healthy, some gastrointestinal conditions, such as IBS, can exacerbate symptoms (Oliveira et al. 2018). For those who are susceptible, fruits that are rich in fermentable oligosaccharides, disaccharides, monosaccharides, and polyols (FODMAPs) may trigger IBS symptoms. Vancamelbeke and Vermeire (2017) have taken their findings to state that some of these fruits are apples, cherries, and stone fruits, where high FODMAPs content is detected and may lead to bloating, cramps, and diarrhea for people with IBS. In order to maintain a balanced diet of fruits while reducing symptoms, a fruit‐based diet short of FODMAPs may be recommended for IBS patients (Vancamelbeke and Vermeire 2017).

## 10 Conclusion and Future Perspectives

In conclusion, there is a lot of evidence supporting the effectiveness of fruit‐based diets and their positive effects on the activity of the immune system, gut health, and immune responses by eating fruits high in fiber, antioxidants, and polyphenols. Fiber‐rich fruits improve immune responses, restrain inflammation, and maintain proper gut flora. More specifically, berries, citrus fruits, apples, and bananas represent significant potential for regulating gut microbiota composition and boosting immunity. Additionally, fruit‐rich diets protect against autoimmune diseases, enhance the body's immune systems against infections, and treat gastrointestinal disorders such as IBD. Fruits such as berries (blueberries, strawberries, raspberries), citrus fruits (oranges, lemons, grapefruit), apples, pears, bananas, pomegranates, papayas, and pineapples are a good source of fiber, polyphenols, and antioxidants to the gut for overall healthiness. A diversity of these foods eaten daily—about two or three servings reduces—inflammation and may help to improve digestion and lead to a balanced gut flora. Every fruit contains unique components that enhance the working of the intestinal barrier, modify microbial populations, and enhance the immune system, making them indispensable for maintaining gut health. Future studies should focus on elaborating the molecular mechanisms by which diets rich in fruits influence gut health, which include the function of specific bioactive compounds and how they interact with gut bacteria. More significant, long‐term clinical research will be needed to investigate further the relationship between consuming fruits and chronic diseases, including autoimmune diseases and gastrointestinal issues.

## Author Contributions

**Sammra Maqsood:** methodology (equal), writing – original draft (equal). **Muhammad Tayyab Arshad:** data curation (equal), writing – review and editing (equal). **Ali Ikram:** supervision (equal), validation (equal). **Kodjo Théodore Gnedeka:** project administration (equal), writing – original draft (equal).

## Consent

This study did not involve humans or animals.

## Conflicts of Interest

The authors declare no conflicts of interest.

## Data Availability

The data supporting this study's findings are available from the corresponding author upon reasonable request.

## References

[fsn370159-bib-0001] Abbas, M. S. , F. Saeed , M. Afzaal , et al. 2022. “Recent Trends in Encapsulation of Probiotics in Dairy and Beverage: A Review.” Journal of Food Processing and Preservation 46, no. 7: e16689. 10.1111/jfpp.16689.

[fsn370159-bib-0002] Abd El‐Aziz, A. H. , K. El‐Sabrout , and M. A. Ghanima . 2024. “Sodium Butyrate Supplementation for Improving Poultry and Rabbit Performance.” Tropical Animal Science Journal 47, no. 2: 252–264. 10.5398/tasj.2024.47.2.252.

[fsn370159-bib-0170] Aburto, N. J. , S. Hanson , H. Gutierrez , L. Hooper , P. Elliott , and F. P. Cappuccio . 2013. “Effect of Increased Potassium Intake on Cardiovascular Risk Factors and Disease: Systematic Review and Meta‐Analyses.” BMJ 346. 10.1136/bmj.f1378 PMC4816263 23558164

[fsn370159-bib-0003] Afzaal, M. , F. Saeed , Y. A. Shah , et al. 2022. “Human Gut Microbiota in Health and Disease: Unveiling the Relationship.” Frontiers in Microbiology 13: 999001. 10.3389/fmicb.2022.999001. 36225386 PMC9549250

[fsn370159-bib-0004] Ahmad, I. Z. , H. Tabassum , A. Ahmad , and M. Kuddus . 2018. “Food Enzymes in Pharmaceutical Industry: Perspectives and Limitations.” In Enzymes in Food Technology: Improvements and Innovations, 41–62. Springer.

[fsn370159-bib-0005] Ajiboye, B. O. , O. A. Ojo , I. Y. Aganzi , et al. 2017. “Antihyperanaemic and Antihyperlipidemic Activities of *Artocarpus altilis* Fruit Based‐Diet on Alloxan‐Induced Diabetic Rats.” International Food Research Journal 24, no. 5: 2133–2139.

[fsn370159-bib-0006] Akuru, E. A. 2022. Effect of Pomegranate (Punica granatum L) Peel Powder Meal on Growth Performance, Gut Integrity, Haemato‐Biochemical Indices, Meat, and Bone Quality of Broiler Chickens (Doctoral dissertation). University of Fort Hare.

[fsn370159-bib-0007] Alami, F. , M. Alizadeh , and K. Shateri . 2022. “The Effect of a Fruit‐Rich Diet on Liver Biomarkers, Insulin Resistance, and Lipid Profile in Patients With Non‐Alcoholic Fatty Liver Disease: A Randomized Clinical Trial.” Scandinavian Journal of Gastroenterology 57, no. 10: 1238–1249. 35710164 10.1080/00365521.2022.2071109

[fsn370159-bib-0008] Alasmar, R. M. , K. Varadharajan , M. Shanmugakonar , and H. A. Al‐Naemi . 2019. “Gut Microbiota and Health: Understanding the Role of Diet.” Food and Nutrition Sciences 10, no. 11: 1344–1373.

[fsn370159-bib-0009] Alissa, E. M. , and G. A. Ferns . 2017. “Dietary Fruits and Vegetables and Cardiovascular Diseases Risk.” Critical Reviews in Food Science and Nutrition 57, no. 9: 1950–1962. 26192884 10.1080/10408398.2015.1040487

[fsn370159-bib-0010] Alonso, V. R. , and F. Guarner . 2013. “Linking the Gut Microbiota to Human Health.” British Journal of Nutrition 109, no. S2: S21–S26. 10.1017/S0007114512005235. 23360877

[fsn370159-bib-0011] Arumugam, T. , C. L. Sona , and M. U. Maheswari . 2021. “Fruits and Vegetables as Superfoods: Scope and Demand.” Journal of Pharmaceutical Innovation 10: 119–129.

[fsn370159-bib-0012] Assimakopoulos, S. F. , C. Triantos , I. Maroulis , and C. Gogos . 2018. “The Role of the Gut Barrier Function in Health and Disease.” Gastroenterology Research 11, no. 4: 261–263. 30116424 10.14740/gr1053w PMC6089582

[fsn370159-bib-0013] Assudani, A. D. 2019. Acceptability Trials of Fructooligosaccharide (FOS) Added Popular Indian Recipes and Impact Evaluation of FOS Intervention in Modulating Gut Microflora, Gut Satietogenic Hormones and Anthropometric Indices of Young Obese Bank Employees of Urban Vadodara: A Fat–Fit Study (Doctoral dissertation). Maharaja Sayajirao University of Baroda.

[fsn370159-bib-0015] Aziz, Q. , J. Doré , A. Emmanuel , F. Guarner , and E. M. M. Quigley . 2013. “Gut Microbiota and Gastrointestinal Health: Current Concepts and Future Directions.” Neurogastroenterology and Motility 25, no. 1: 4–15. 23279728 10.1111/nmo.12046

[fsn370159-bib-0016] Bäckhed, F. , C. M. Fraser , Y. Ringel , et al. 2012. “Defining a Healthy Human Gut Microbiome: Current Concepts, Future Directions, and Clinical Applications.” Cell Host & Microbe 12, no. 5: 611–622. 10.1016/j.chom.2012.10.012. 23159051

[fsn370159-bib-0156] Bai, M. , H. Liu , Y. Yan , et al. 2024. “Hydrolyzed Protein Formula Improves the Nutritional Tolerance by Increasing Intestinal Development and Altering Cecal Microbiota in Low‐Birth‐Weight Piglets.” Frontiers in Nutrition 11: 1439110. 10.3389/fnut.2024.1439110. 39555191 PMC11565607

[fsn370159-bib-0174] Bandelow, B. , and S. Michaelis . 2015. “Epidemiology of Anxiety Disorders in the 21st Century.” Dialogues in Clinical Neuroscience 17, no. 3: 327–335. 26487813 10.31887/DCNS.2015.17.3/bbandelow PMC4610617

[fsn370159-bib-0017] Barreca, D. , G. Mandalari , A. Calderaro , et al. 2020. “Citrus Flavones: An Update on Sources, Biological Functions, and Health Promoting Properties.” Plants 9, no. 3: 288. 32110931 10.3390/plants9030288 PMC7154817

[fsn370159-bib-0018] Beigoli, S. , and M. H. Boskabady . 2024. “The Molecular Basis of the Immunomodulatory Effects of Natural Products: A Comprehensive Review.” Phytomedicine 135: 156028. 10.1016/j.phymed.2024.156028. 39276685

[fsn370159-bib-0019] Bellavite, P. , and A. Donzelli . 2020. “Hesperidin and SARS‐CoV‐2: New Light on the Healthy Function of Citrus Fruits.” Antioxidants 9, no. 8: 742. 32823497 10.3390/antiox9080742 PMC7465267

[fsn370159-bib-0020] Beukema, M. , M. M. Faas , and P. de Vos . 2020. “The Effects of Different Dietary Fiber Pectin Structures on the Gastrointestinal Immune Barrier: Impact via Gut Microbiota and Direct Effects on Immune Cells.” Experimental & Molecular Medicine 52, no. 9: 1364–1376. 32908213 10.1038/s12276-020-0449-2 PMC8080816

[fsn370159-bib-0022] Blanco‐Pérez, F. , H. Steigerwald , S. Schülke , S. Vieths , M. Toda , and S. Scheurer . 2021. “The Dietary Fiber Pectin: Health Benefits and Potential for the Treatment of Allergies by Modulation of Gut Microbiota.” Current Allergy and Asthma Reports 21: 1–19. 10.1007/s11882-021-01020-z PMC8433104 34505973

[fsn370159-bib-0165] Bohn, L. R. , A. P. Dresch , D. Manica , et al. 2024. “Antiproliferative Effect of Phenolic Compounds Extracted from Winery Pomace on TPC‐1 Thyroid Cancer Cells.” Food Bioscience 60: 104457.

[fsn370159-bib-0023] Bouyahya, A. , N. E. Omari , N. El Hachlafi , et al. 2022. “Chemical Compounds of Berry‐Derived Polyphenols and Their Effects on Gut Microbiota, Inflammation, and Cancer.” Molecules 27, no. 10: 3286. 10.3390/molecules27103286. 35630763 PMC9146061

[fsn370159-bib-0025] Cénit, M. C. , V. Matzaraki , E. F. Tigchelaar , and A. Zhernakova . 2014. “Rapidly Expanding Knowledge on the Role of the Gut Microbiome in Health and Disease.” Biochimica et Biophysica Acta (BBA) ‐ Molecular Basis of Disease 1842, no. 10: 1981–1992. 10.1016/j.bbadis.2014.05.023. 24882755

[fsn370159-bib-0153] Chen, F. , Y. Wang , K. Wang , et al. 2023. “Effects of *Litsea cubeba* Essential Oil on Growth Performance, Blood Antioxidation, Immune Function, Apparent Digestibility of Nutrients, and Fecal Microflora of Pigs.” Frontiers in Pharmacology 14: 1166022. 10.3389/fphar.2023.1166022. 37465523 PMC10350539

[fsn370159-bib-0026] Chen, J. , Y. Shu , Y. Chen , et al. 2022. “Evaluation of Antioxidant Capacity and Gut Microbiota Modulatory Effects of Different Kinds of Berries.” Antioxidants 11: 1020. 35624885 10.3390/antiox11051020 PMC9137550

[fsn370159-bib-0027] Chong, C. W. , M. S. Liew , W. Ooi , et al. 2024. “Effect of Green Banana and Pineapple Fibre Powder Consumption on Host Gut Microbiome.” Frontiers in Nutrition 11: 1437645. 10.3389/fnut.2024.1437645. 39246394 PMC11378528

[fsn370159-bib-0028] Conlon, M. A. , and A. R. Bird . 2014. “The Impact of Diet and Lifestyle on Gut Microbiota and Human Health.” Nutrients 7, no. 1: 17–44. 10.3390/nu7010017. 25545101 PMC4303825

[fsn370159-bib-0029] Coutinho‐Wolino, K. S. , M. F. Melo , J. C. Mota , D. Mafra , J. T. Guimarães , and M. B. Stockler‐Pinto . 2024. “Blueberry, Cranberry, Raspberry, and Strawberry as Modulators of the Gut Microbiota: Target for Treatment of Gut Dysbiosis in Chronic Kidney Disease? From Current Evidence to Future Possibilities.” Nutrition Reviews 82, no. 2: 248–261. 10.1093/nutrit/nuad048. 37164634

[fsn370159-bib-0030] Crowe‐White, K. , C. E. O'Neil , J. S. Parrott , et al. 2016. “Impact of 100% Fruit Juice Consumption on Diet and Weight Status of Children: An Evidence‐Based Review.” Critical Reviews in Food Science and Nutrition 56, no. 5: 871–884. 10.1080/10408398.2015.1061475. 26091353

[fsn370159-bib-0178] Crupi, P. , M. F. Faienza , M. Y. Naeem , F. Corbo , M. L. Clodoveo , and M. Muraglia . 2023. “Overview of the Potential Beneficial Effects of Carotenoids on Consumer Health and Well‐Being.” Antioxidants 12, no. 5: 1069. 37237935 10.3390/antiox12051069 PMC10215867

[fsn370159-bib-0031] Dahiya, D. , and P. S. Nigam . 2022. “Nutrition and Health Through the Use of Probiotic Strains in Fermentation to Produce Non‐Dairy Functional Beverage Products Supporting Gut Microbiota.” Food 11, no. 18: 2760. 10.3390/foods11182760 PMC9497984 36140888

[fsn370159-bib-0032] Das, B. , and G. B. Nair . 2019. “Homeostasis and Dysbiosis of the Gut Microbiome in Health and Disease.” Journal of Biosciences 44, no. 5: 117. 10.1007/s12038-019-9926-y. 31719226

[fsn370159-bib-0033] de Andra, L. S. , F. A. H. Sarda , N. B. F. Pereira , et al. 2021. “Effect of Unripe Banana Flour on Gut‐Derived Uremic Toxins in Individuals Undergoing Peritoneal Dialysis: A Randomized, Double‐Blind, Placebo‐Controlled, Crossover Trial.” Nutrients 13, no. 2: 646. 10.3390/nu13020646. 33671166 PMC7922008

[fsn370159-bib-0167] de Souza, B. M. S. , T. F. Borgonovi , S. N. Casarotti , S. D. Todorov , and A. L. B. Penna . 2019. “ *Lactobacillus casei* and *Lactobacillus fermentum* Strains Isolated from Mozzarella Cheese: Probiotic Potential, Safety, Acidifying Kinetic Parameters and Viability Under Gastrointestinal Tract Conditions.” Probiotics and Antimicrobial Proteins 11: 382–396. 29542032 10.1007/s12602-018-9406-y

[fsn370159-bib-0035] Donadio, J. L. , J. P. Fabi , M. B. Sztein , and R. Salerno‐Gonçalves . 2024. “Dietary Fiber Pectin: Challenges and Potential Anti‐Inflammatory Benefits for Preterms and Newborns.” Frontiers in Nutrition 10: 1286138. 10.3389/fnut.2023.1286138. 38283907 PMC10811139

[fsn370159-bib-0148] Dong, L. , F. Dong , P. Guo , et al. 2025. “Gut Microbiota as a New Target for Hyperuricemia: A Perspective from Natural Plant Products.” Phytomedicine 138: 156402. 10.1016/j.phymed.2025.156402. 39874797

[fsn370159-bib-0036] Doriya, K. , D. S. Kumar , and B. N. Thorat . 2022. “A Systematic Review on Fruit‐Based Fermented Foods as an Approach to Improve Dietary Diversity.” Journal of Food Processing and Preservation 46, no. 11: e16994. 10.1111/jfpp.16994.

[fsn370159-bib-0037] Dreher, M. L. 2018. “Whole Fruits and Fruit Fiber Emerging Health Effects.” Nutrients 10, no. 12: 1833. 10.3390/nu10121833. 30487459 PMC6315720

[fsn370159-bib-0039] El‐Sabrout, K. , S. Landolfi , and F. Ciani . 2024. “Feed Additives and Enrichment Materials to Reduce Chicken Stress, Maximize Productivity, and Improve Welfare.” Veterinary World 17, no. 9: 2044–2052. 39507789 10.14202/vetworld.2024.2044-2052 PMC11536731

[fsn370159-bib-0040] Elshahed, M. S. , A. Miron , A. C. Aprotosoaie , and M. A. Farag . 2021. “Pectin in Diet: Interactions With the Human Microbiome, Role in Gut Homeostasis, and Nutrient‐Drug Interactions.” Carbohydrate Polymers 255: 117388. 33436217 10.1016/j.carbpol.2020.117388

[fsn370159-bib-0157] Fan, J. , L. Wang , T. Yang , et al. 2024. “Comparative Analysis of Gut Microbiota in Incident and Prevalent Peritoneal Dialysis Patients with Peritoneal Fibrosis, Correlations with Peritoneal Equilibration Test Data in the Peritoneal Fibrosis Cohort.” Therapeutic Apheresis and Dialysis. 10.1111/1744-9987.14226. PMC13540531 39520210

[fsn370159-bib-0041] Fardet, A. , and C. Richonnet . 2020. “Nutrient Density and Bioaccessibility, and the Antioxidant, Satiety, Glycemic, and Alkalinizing Potentials of Fruit‐Based Foods According to the Degree of Processing: A Narrative Review.” Critical Reviews in Food Science and Nutrition 60, no. 19: 3233–3258. 31674823 10.1080/10408398.2019.1682512

[fsn370159-bib-0161] Fazio, S. , P. Bellavite , E. Zanolin , P. A. McCullough , S. Pandolfi , and F. Affuso . 2021. “Retrospective Study of Outcomes and Hospitalization Rates of Patients in Italy with a Confirmed Diagnosis of Early COVID‐19 and Treated at Home Within 3 Days or After 3 Days of Symptom Onset with Prescribed and Non‐Prescribed Treatments Between November 2020 and August 2021.” Medical Science Monitor: international medical journal of experimental and clinical research 27: e935379–e935371. 34966165 10.12659/MSM.935379 PMC8725339

[fsn370159-bib-0042] Fouhse, J. M. , R. T. Zijlstra , and B. P. Willing . 2016. “The Role of Gut Microbiota in the Health and Disease of Pigs.” Animal Frontiers 6, no. 3: 30–36.

[fsn370159-bib-0043] Fulton, S. L. , M. C. McKinley , I. S. Young , C. R. Cardwell , and J. V. Woodside . 2016. “The Effect of Increasing Fruit and Vegetable Consumption on Overall Diet: A Systematic Review and Meta‐Analysis.” Critical Reviews in Food Science and Nutrition 56, no. 5: 802–816. 25118067 10.1080/10408398.2012.727917

[fsn370159-bib-0044] Gâtlan, A. M. , and G. Gutt . 2021. “Sea Buckthorn in Plant Based Diets. An Analytical Approach of Sea Buckthorn Fruits Composition: Nutritional Value, Applications, and Health Benefits.” International Journal of Environmental Research and Public Health 18, no. 17: 8986. 10.3390/ijerph18178986. 34501575 PMC8431556

[fsn370159-bib-0045] Giangrieco, I. , M. A. Ciardiello , M. Tamburrini , et al. 2023. “Comparative Analysis of the Immune Response and the Clinical Allergic Reaction to Papain‐Like Cysteine Proteases From Fig, Kiwifruit, Papaya, Pineapple and Mites in an Italian Population.” Food 12, no. 15: 2852. 10.3390/foods12152852 PMC10417190 37569122

[fsn370159-bib-0168] Gibson, G. R. , R. Hutkins , M. E. Sanders , et al. 2017. “Expert Consensus Document: The International Scientific Association for Probiotics and Prebiotics (ISAPP) Consensus Statement on the Definition and Scope of Prebiotics.” Nature Reviews Gastroenterology & Hepatology 14, no. 8: 491–502. 28611480 10.1038/nrgastro.2017.75

[fsn370159-bib-0046] Gomes, I. A. , A. Venâncio , J. P. Lima , and O. Freitas‐Silva . 2021. “Fruit‐Based Non‐Dairy Beverage: A New Approach for Probiotics.” Advances in Biological Chemistry 11, no. 6: 302–330.

[fsn370159-bib-0047] Govers, C. , M. Berkel Kasikci , A. A. van der Sluis , and J. J. Mes . 2018. “Review of the Health Effects of Berries and Their Phytochemicals on the Digestive and Immune Systems.” Nutrition Reviews 76, no. 1: 29–46. 10.1093/nutrit/nux039. 29087531

[fsn370159-bib-0048] Greenhalgh, K. , K. M. Meyer , K. M. Aagaard , and P. Wilmes . 2016. “The Human Gut Microbiome in Health: Establishment and Resilience of Microbiota Over a Lifetime.” Environmental Microbiology 18, no. 7: 2103–2116. 27059297 10.1111/1462-2920.13318 PMC7387106

[fsn370159-bib-0049] Henning, S. M. , J. Yang , P. Shao , et al. 2017. “Health Benefit of Vegetable/Fruit Juice‐Based Diet: Role of Microbiome.” Scientific Reports 7, no. 1: 2167. 10.1038/s41598-017-02200-6. 28526852 PMC5438379

[fsn370159-bib-0050] Hikawczuk, T. M. , P. Wróblewska , A. Szuba‐Trznadel , A. Rusiecka , A. Zinchuk , and K. Laszki‐Szczachor . 2024. “Pectin and Pectin Oligosaccharides Obtained From Agro‐Wastes as a Constituents of Soluble Dietary Fibre: Effect on the Stabili‐Zation of Intestinal Microbiome and Immunity of Humans and Animals–A Review.”

[fsn370159-bib-0051] Hollister, E. B. , C. Gao , and J. Versalovic . 2014. “Compositional and Functional Features of the Gastrointestinal Microbiome and Their Effects on Human Health.” Gastroenterology 146, no. 6: 1449–1458. 10.1053/j.gastro.2014.01.052. 24486050 PMC4181834

[fsn370159-bib-0052] Hooper, L. V. , D. R. Littman , and A. J. Macpherson . 2012. “Interactions Between the Microbiota and the Immune System.” Science 336, no. 6086: 1268–1273. 22674334 10.1126/science.1223490 PMC4420145

[fsn370159-bib-0054] Hunter, D. C. , M. A. Skinner , and A. R. Ferguson . 2016. “Kiwifruit and Health.” In Fruits, Vegetables, and Herbs, 239–269. Academic Press.

[fsn370159-bib-0056] Ikram, A. , S. Z. Safdar , M. T. Arshad , A. Rasheed , and K. T. Gnedeka . 2024. “An Overview of Postbiotics: Unveiling Their Distinct Role in Gut Health.” Food and Agricultural Immunology 35, no. 1: 2434463. 10.1080/09540105.2024.2434463.

[fsn370159-bib-0055] Ikram, A. , W. Khalid , M. A. Rahim , et al. 2021. “Foods Influence the Gut Microbiota: Boost Immunity Against Covid‐19.” Acta Scientific Nutritional Health 5, no. 12: 598.

[fsn370159-bib-0057] Jaiswal, A. K. , ed. 2020. Nutritional Composition and Antioxidant Properties of Fruits and Vegetables. Academic Press.

[fsn370159-bib-0177] Jakubowicz, D. , Y. Matz , Z. Landau , et al. 2024. “Interaction Between Early Meals (Big‐Breakfast Diet), Clock Gene mRNA Expression, and Gut Microbiome to Regulate Weight Loss and Glucose Metabolism in Obesity and Type 2 Diabetes.” International Journal of Molecular Sciences 25, no. 22: 12355. 39596418 10.3390/ijms252212355 PMC11594859

[fsn370159-bib-0058] Jhee, J. H. , Y. K. Kee , J. T. Park , et al. 2019. “A Diet Rich in Vegetables and Fruit and Incident CKD: A Community‐Based Prospective Cohort Study.” American Journal of Kidney Diseases 22: 479. 10.1053/j.ajkd.2019.02.023 31040089

[fsn370159-bib-0060] Katsirma, Z. , E. Dimidi , A. Rodriguez‐Mateos , and K. Whelan . 2021. “Fruits and Their Impact on the Gut Microbiota, Gut Motility and Constipation.” Food & Function 12, no. 19: 8850–8866. 34505614 10.1039/d1fo01125a

[fsn370159-bib-0062] Kogut, M. H. , Y. XiaoNan , Y. JianMin , and L. J. C. R. Broom . 2017. “Gut Health in Poultry.” CABI Reviews 25: 1–7.

[fsn370159-bib-0064] Komarnytsky, S. , C. Wagner , J. Gutierrez , and O. M. Shaw . 2023. “Berries in Microbiome‐Mediated Gastrointestinal, Metabolic, and Immune Health.” Current Nutrition Reports 12, no. 1: 151–166. 36738429 10.1007/s13668-023-00449-0

[fsn370159-bib-0066] Komati, N. , F. Vieux , M. Maillot , et al. 2024b. “Environmental Impact and Nutritional Quality of Adult Diet in France Based on Fruit and Vegetable Intakes.” European Journal of Nutrition 63, no. 1: 195–207. 10.1007/s00394-023-03252-3. 37801156 PMC10799092

[fsn370159-bib-0065] Komati, N. , J. P. Cravedi , J. M. Lecerf , et al. 2024a. “Potential Health Benefits of a Diet Rich in Organic Fruit and Vegetables Versus a Diet Based on Conventional Produce: A Systematic Review.” Nutrition Reviews 83, no. 3: nuae104. 10.1093/nutrit/nuae104. PMC11819487 39101594

[fsn370159-bib-0067] Koutsos, A. , K. M. Tuohy , and J. A. Lovegrove . 2015. “Apples and Cardiovascular Health—Is the Gut Microbiota a Core Consideration?” Nutrients 7, no. 6: 3959–3998. 26016654 10.3390/nu7063959 PMC4488768

[fsn370159-bib-0179] Krawczyk, K. , A. Szabelska‐Beręsewicz , S. W. Przemieniecki , M. Szymańczyk , and A. Obrępalska‐Stęplowska . 2022. “Insect Gut Bacteria Promoting the Growth of Tomato Plants (*Solanum lycopersicum* L.).” International Journal of Molecular Sciences 23, no. 21: 13548. 36362334 10.3390/ijms232113548 PMC9657159

[fsn370159-bib-0068] Lakshmanan, A. P. , A. Mingione , F. Pivari , et al. 2022. “Modulation of Gut Microbiota: The Effects of a Fruits and Vegetables Supplement.” Frontiers in Nutrition 9: 930883. 10.3389/fnut.2022.930883. 36211488 PMC9537686

[fsn370159-bib-0070] Lavefve, L. , L. R. Howard , and F. Carbonero . 2020. “Berry Polyphenols Metabolism and Impact on Human Gut Microbiota and Health.” Food & Function 11, no. 1: 45–65. 31808762 10.1039/c9fo01634a

[fsn370159-bib-0072] Lebaka, V. R. , Y. J. Wee , W. Ye , and M. Korivi . 2021. “Nutritional Composition and Bioactive Compounds in Three Different Parts of Mango Fruit.” International Journal of Environmental Research and Public Health 18, no. 2: 741. 33467139 10.3390/ijerph18020741 PMC7830918

[fsn370159-bib-0073] Lepaus, B. M. , B. S. Valiati , B. G. Machado , et al. 2023. “Impact of Ultrasound Processing on the Nutritional Components of Fruit and Vegetable Juices.” Trends in Food Science and Technology 138: 752–765. 10.1016/j.tifs.2023.07.002.

[fsn370159-bib-1073] Li, B. , Q. Gao , Y. Wu , et al. 2025. “Broad‐spectrum anti‐inflammatory and antioxidant therapy of inflammatory‐storm actuated diseases via programmable self‐derived cryo‐dead neutrophils.” Chemical Engineering Journal 507: 160643. 10.1016/j.cej.2025.160643.

[fsn370159-bib-0146] Liang, M. , T. Li , Y. Qu , et al. 2023. “Mitigation Mechanism of Resveratrol on Thermally Induced Trans‐α‐Linolenic Acid of Trilinolenin.” LWT 189: 115508. 10.1016/j.lwt.2023.115508.

[fsn370159-bib-0075] Liu, R. H. 2013. “Health‐Promoting Components of Fruits and Vegetables in the Diet.” Advances in Nutrition 4, no. 3: 384S–392S. 23674808 10.3945/an.112.003517 PMC3650511

[fsn370159-bib-0147] Liu, W. , Y. Han , J. An , et al. 2025a. “Alternation in Sequence Features and Their Influence on the Anti‐Inflammatory Activity of Soy Peptides During Digestion and Absorption in Different Enzymatic Hydrolysis Conditions.” Food Chemistry 471: 142824. 10.1016/j.foodchem.2025.142824. 39799691

[fsn370159-bib-0154] Liu, Y. , Y. Wang , Y. Fu , et al. 2025b. “Fabrication of Temperature and pH Dual‐Sensitive Semi‐Interpenetrating Network Hydrogel with Enhanced Adhesion and Antibacterial Properties.” Polymer 326: 128343. 10.1016/j.polymer.2025.128343.

[fsn370159-bib-0076] Lu, X. , C. Zhao , H. Shi , et al. 2023. “Nutrients and Bioactives in Citrus Fruits: Different Citrus Varieties, Fruit Parts, and Growth Stages.” Critical Reviews in Food Science and Nutrition 63, no. 14: 2018–2041. 10.1080/10408398.2021.1969891. 34609268

[fsn370159-bib-0172] Ma, R. , X. Zhao , S. H. Ho , et al. 2020. “Co‐Production of Lutein and Fatty Acid in Microalga *Chlamydomonas* sp. JSC4 in Response to Different Temperatures with Gene Expression Profiles.” Algal Research 47: 101821.

[fsn370159-bib-0077] Maldonado‐Celis, M. E. , E. M. Yahia , R. Bedoya , et al. 2019. “Chemical Composition of Mango (*Mangifera indica* L.) Fruit: Nutritional and Phytochemical Compounds.” Frontiers in Plant Science 10: 1073. 10.3389/fpls.2019.01073. 31681339 PMC6807195

[fsn370159-bib-0078] Marino, M. , S. Venturi , G. Gargari , et al. 2024. “Berries‐Gut Microbiota Interaction and Impact on Human Health: A Systematic Review of Randomized Controlled Trials.” Food Reviews International 40, no. 9: 2618–2640. 10.1080/87559129.2023.2276765.

[fsn370159-bib-0079] Maugeri, A. , S. Cirmi , P. L. Minciullo , et al. 2019. “Citrus Fruits and Inflammaging: A Systematic Review.” Phytochemistry Reviews 18: 1025–1049.

[fsn370159-bib-0081] McMacken, M. , and S. Shah . 2017. “A Plant‐Based Diet for the Prevention and Treatment of Type 2 Diabetes.” Journal of Geriatric Cardiology 14, no. 5: 342–354. 28630614 10.11909/j.issn.1671-5411.2017.05.009 PMC5466941

[fsn370159-bib-0082] Meena, N. K. , K. Choudhary , N. Negi , V. S. Meena , and V. Gupta . 2021. “Nutritional Composition of Stone Fruits.” Production Technology of Stone Fruits 20: 227–251.

[fsn370159-bib-0163] Meyer, N. , O. Saburow , M. Hohberg , A. N. Hrymak , F. Henning , and L. Kärger . 2020. “Parameter Identification of Fiber Orientation Models Based on Direct Fiber Simulation with Smoothed Particle Hydrodynamics.” Journal Of Composites Science 4, no. 2: 77.

[fsn370159-bib-0084] Miles, E. A. , and P. C. Calder . 2021. “Effects of Citrus Fruit Juices and Their Bioactive Components on Inflammation and Immunity: A Narrative Review.” Frontiers in Immunology 12: 712608. 10.3389/fimmu.2021.712608. 34249019 PMC8264544

[fsn370159-bib-0085] Mishra, S. K. , P. M. Ishfaq , S. Tripathi , and N. Gupta . 2022. “Fruits as Boosters of the Immune System.” In Plants and Phytomolecules for Immunomodulation: Recent Trends and Advances, 391–411. Springer Nature Singapore.

[fsn370159-bib-0175] O'Mahony, S. M. , K. A. McVey Neufeld , R. V. Waworuntu , et al. 2020. “The Enduring Effects of Early‐Life Stress on the Microbiota–Gut–Brain Axis Are Buffered by Dietary Supplementation with Milk Fat Globule Membrane and a Prebiotic Blend.” European Journal of Neuroscience 51, no. 4: 1042–1058. 31339598 10.1111/ejn.14514

[fsn370159-bib-0086] Olazcuaga, L. , R. Baltenweck , N. Leménager , et al. 2023. “Metabolic Consequences of Various Fruit‐Based Diets in a Generalist Insect Species.” eLife 12: e84370. 10.7554/eLife.84370. 37278030 PMC10259468

[fsn370159-bib-0087] Oliveira, A. , A. L. Amaro , and M. Pintado . 2018. “Impact of Food Matrix Components on Nutritional and Functional Properties of Fruit‐Based Products.” Current Opinion in Food Science 22: 153–159. 10.1016/j.cofs.2018.04.002.

[fsn370159-bib-0088] Olivier, R. 2015. “An Update on the Use of Proteolytic Enzymes as a Means to Reduce Inflammation and Pain.” Original Internist 22, no. 2: 300–304.

[fsn370159-bib-0089] Pap, N. , M. Fidelis , L. Azevedo , et al. 2021. “Berry Polyphenols and Human Health: Evidence of Antioxidant, Anti‐Inflammatory, Microbiota Modulation, and Cell‐Protecting Effects.” Current Opinion in Food Science 42: 167–186. 10.1016/j.cofs.2021.06.003.

[fsn370159-bib-0090] Patil, B. S. , G. K. Jayaprakasha , and K. N. C. Murthy . 2017. “Beyond Vitamin C: The Diverse, Complex Health‐Promoting Properties of Citrus Fruits.” Citrus Research & Technology 38, no. 1: 107–121. 10.4322/crt.ICC063.

[fsn370159-bib-0091] Peluso, I. , and M. Palmery . 2014. “Risks of Misinterpretation in the Evaluation of the Effect of Fruit‐Based Drinks in Postprandial Studies.” Gastroenterology Research and Practice 2014, no. 1: 870547. 25610461 10.1155/2014/870547 PMC4295616

[fsn370159-bib-0092] Pluske, J. R. , D. L. Turpin , and J. C. Kim . 2018. “Gastrointestinal Tract (Gut) Health in the Young Pig.” Animal Nutrition 4, no. 2: 187–196. 30140758 10.1016/j.aninu.2017.12.004 PMC6104527

[fsn370159-bib-0093] Rajoka, M. S. R. , J. Shi , H. M. Mehwish , et al. 2017. “Interaction Between Diet Composition and Gut Microbiota and Its Impact on Gastrointestinal Tract Health.” Food Science and Human Wellness 6, no. 3: 121–130. 10.1016/j.fshw.2017.07.003.

[fsn370159-bib-0095] Rezende, E. S. V. , G. C. Lima , and M. M. V. Naves . 2021. “Dietary Fibers as Beneficial Microbiota Modulators: A Proposed Classification by Prebiotic Categories.” Nutrition 89: 111217. 33838493 10.1016/j.nut.2021.111217

[fsn370159-bib-0096] Rist, V. T. S. , E. Weiss , M. Eklund , and R. Mosenthin . 2013. “Impact of Dietary Protein on Microbiota Composition and Activity in the Gastrointestinal Tract of Piglets in Relation to Gut Health: A Review.” Animal 7, no. 7: 1067–1078. 10.1017/S1751731113000062. 23410993

[fsn370159-bib-0098] Rodríguez, L. G. R. , V. M. Z. Gasga , M. Pescuma , C. Van Nieuwenhove , F. Mozzi , and J. A. S. Burgos . 2021. “Fruits and Fruit Byproducts as Sources of Bioactive Compounds. Benefits and Trends of Lactic Acid Fermentation in the Development of Novel Fruit‐Based Functional Beverages.” Food Research International 140: 109854. 33648172 10.1016/j.foodres.2020.109854

[fsn370159-bib-0099] Saini, R. K. , A. Ranjit , K. Sharma , et al. 2022. “Bioactive Compounds of Citrus Fruits: A Review of Composition and Health Benefits of Carotenoids, Flavonoids, Limonoids, and Terpenes.” Antioxidants 11, no. 2: 239. 35204122 10.3390/antiox11020239 PMC8868476

[fsn370159-bib-0164] Salehi, A. , S. Emami , M. Keighobadi , and H. Mirzaei . 2019. “An Overview of the Effects of Polyphenols on Cardiac Mitochondrial Function.” Journal of Mazandaran University of Medical Sciences 28, no. 170: 211–224.

[fsn370159-bib-0100] Sanofer, A. A. 2014. “Role of Citrus Fruits in Health.” Journal of Pharmaceutical Sciences and Research 6, no. 2: 121.

[fsn370159-bib-0101] Santacroce, L. , L. Bottalico , I. A. Charitos , et al. 2024. “Exploitation of Natural Byproducts for the Promotion of Healthy Outcomes in Humans: Special Focus on Antioxidant and Anti‐Inflammatory Mechanisms and Modulation of the Gut Microbiota.” Antioxidants 13, no. 7: 796. 39061865 10.3390/antiox13070796 PMC11273986

[fsn370159-bib-0102] Santos, G. M. G. C. D. , A. M. R. Silva , W. O. D. Carvalho , C. R. Rech , and M. R. Loch . 2019. “Perceived Barriers for the Consumption of Fruits and Vegetables in Brazilian Adults.” Ciência & Saúde Coletiva 24, no. 7: 2461–2470. 10.1590/1413-81232018247.19992017. 31340265

[fsn370159-bib-0103] Sanz, Y. , M. Olivares , Á. Moya‐Pérez , and C. Agostoni . 2015. “Understanding the Role of Gut Microbiome in Metabolic Disease Risk.” Pediatric Research 77, no. 1: 236–244. 10.1038/pr.2014.170. 25314581

[fsn370159-bib-0105] Sarrimanolis, E. 2023. Bromelain as a Feed Additive to Promote Broiler Performance Nutrient Digestibility and Health (Master's Thesis). University of Pretoria.

[fsn370159-bib-0106] Satija, A. , and F. B. Hu . 2018. “Plant‐Based Diets and Cardiovascular Health.” Trends in Cardiovascular Medicine 28, no. 7: 437–441. 29496410 10.1016/j.tcm.2018.02.004 PMC6089671

[fsn370159-bib-0107] Sauceda, A. E. Q. , R. Pacheco‐Ordaz , J. F. Ayala‐Zavala , et al. 2017. “Impact of Fruit Dietary Fibers and Polyphenols on Modulation of the Human Gut Microbiota.” Fruit and Vegetable Phytochemicals: Chemistry and Human Health 20: 405–422.

[fsn370159-bib-0108] Septembre‐Malaterre, A. , F. Remize , and P. Poucheret . 2018. “Fruits and Vegetables, as a Source of Nutritional Compounds and Phytochemicals: Changes in Bioactive Compounds During Lactic Fermentation.” Food Research International 104: 86–99. 29433787 10.1016/j.foodres.2017.09.031

[fsn370159-bib-0109] Sherwin, E. , T. G. Dinan , and J. F. Cryan . 2018. “Recent Developments in Understanding the Role of the Gut Microbiota in Brain Health and Disease.” Annals of the New York Academy of Sciences 1420, no. 1: 5–25. 28768369 10.1111/nyas.13416

[fsn370159-bib-0110] Silva‐ López, R. E. , and R. N. Gonçalves . 2019. “Therapeutic Proteases From Plants: Biopharmaceuticals With Multiple Applications.” Journal of Applied Biology & Biotechnology 6, no. 2: 101–109. 10.15406/jabb.2019.06.00180.

[fsn370159-bib-0111] Singh, D. N. , J. S. Bohra , T. P. Dubey , et al. 2023. “Common Foods for Boosting Human Immunity: A Review.” Food Science & Nutrition 11, no. 11: 6761–6774. 37970422 10.1002/fsn3.3628 PMC10630845

[fsn370159-bib-0112] Sireswar, S. , G. Dey , and S. Biswas . 2021. “Influence of Fruit‐Based Beverages on Efficacy of Lacticaseibacillus Rhamnosus GG (*Lactobacillus rhamnosus* GG) Against DSS‐Induced Intestinal Inflammation.” Food Research International 149: 110661. 10.1016/j.foodres.2021.110661. 34600663

[fsn370159-bib-0180] Sivakanthan, S. , S. Fawzia , T. Madhujith , and A. Karim . 2022. “Synergistic effects of oleogelators in tailoring the properties of oleogels: A review.” Comprehensive Reviews in Food Science and Food Safety 21, no. 4: 3507–3539. 35591753 10.1111/1541-4337.12966

[fsn370159-bib-0162] Slavin, J. 2013. “Fiber and Prebiotics: Mechanisms and Health Benefits.” Nutrients 5, no. 4: 1417–1435. 23609775 10.3390/nu5041417 PMC3705355

[fsn370159-bib-0114] Soory, M. 2009. “Relevance of Nutritional Antioxidants in Metabolic Syndrome, Ageing and Cancer: Potential for Therapeutic Targeting.” Infectious Disorders‐Drug Targets (Formerly Current Drug Targets‐Infectious Disorders) 9, no. 4: 400–414. 10.2174/187152609788922537. 19689382

[fsn370159-bib-0117] Stuhl, C. , L. Cicero , J. Sivinski , et al. 2011. “Longevity of Multiple Species of Tephritid (Diptera) Fruit Fly Parasitoids (Hymenoptera: Braconidae: Opiinae) Provided Exotic and Sympatric‐Fruit Based Diets.” Journal of Insect Physiology 57, no. 11: 1463–1470. 21839085 10.1016/j.jinsphys.2011.07.015

[fsn370159-bib-0118] Sun‐Waterhouse, D. 2011. “The Development of Fruit‐Based Functional Foods Targeting the Health and Wellness Market: A Review.” International Journal of Food Science & Technology 46, no. 5: 899–920.

[fsn370159-bib-0171] Tan, C. , J. Xue , S. Abbas , B. Feng , X. Zhang , and S. Xia . 2014. “Liposome as a Delivery System for Carotenoids: Comparative Antioxidant Activity of Carotenoids as Measured by Ferric Reducing Antioxidant Power, DPPH Assay and Lipid Peroxidation.” Journal of Agricultural and Food Chemistry 62, no. 28: 6726–6735. 24745755 10.1021/jf405622f

[fsn370159-bib-0120] Tapsell, L. C. , E. P. Neale , A. Satija , and F. B. Hu . 2016. “Foods, Nutrients, and Dietary Patterns: Interconnections and Implications for Dietary Guidelines.” Advances in Nutrition 7, no. 3: 445–454. 27184272 10.3945/an.115.011718 PMC4863273

[fsn370159-bib-0173] Totaro, M. , F. Castellani , F. Di Serafino , et al. 2023. “Role of Environmental Sanitization in Health Clinics: Evaluation of Potassium Peroxymonosulfate (KMPS) Efficacy at Two Different Concentrations.” Igiene e Sanità Pubblica 81, no. 1. 36749592

[fsn370159-bib-0122] Tufail, T. , H. Bader Ul Ain , S. Noreen , A. Ikram , M. T. Arshad , and M. A. Abdullahi . 2024. “Nutritional Benefits of Lycopene and Beta‐Carotene: A Comprehensive Overview.” Food Science & Nutrition 12, no. 11: 8715–8741. 39619948 10.1002/fsn3.4502 PMC11606860

[fsn370159-bib-0123] Uraipan, S. , P. Brigidi , and T. Hongpattarakere . 2014. “Antagonistic Mechanisms of Synbiosis Between *Lactobacillus plantarum* CIF17AN2 and Green Banana Starch in the Proximal Colon Model Challenged With *Salmonella Typhimurium* .” Anaerobe 28: 44–53. 24858321 10.1016/j.anaerobe.2014.05.002

[fsn370159-bib-0125] Vancamelbeke, M. , and S. Vermeire . 2017. “The Intestinal Barrier: A Fundamental Role in Health and Disease.” Expert Review of Gastroenterology & Hepatology 11, no. 9: 821–834. 28650209 10.1080/17474124.2017.1343143 PMC6104804

[fsn370159-bib-0127] Venter, C. 2023. “Immunonutrition: Diet Diversity, Gut Microbiome and Prevention of Allergic Diseases.” Allergy, Asthma & Immunology Research 15, no. 5: 545–561. 10.4168/aair.2023.15.5.545 PMC10570780 37827976

[fsn370159-bib-0128] Vincente, A. R. , G. A. Manganaris , C. M. Ortiz , G. O. Sozzi , and C. H. Crisosto . 2014. “Nutritional Quality of Fruits and Vegetables.” In Postharvest Handling, 69–122. Academic press.

[fsn370159-bib-0129] Vitetta, L. , D. Briskey , H. Alford , S. Hall , and S. Coulson . 2014. “Probiotics, Prebiotics and the Gastrointestinal Tract in Health and Disease.” Inflammopharmacology 22: 135–154. 24633989 10.1007/s10787-014-0201-4

[fsn370159-bib-0131] von Martels, J. Z. , M. S. Sadabad , A. R. Bourgonje , et al. 2017. “The Role of Gut Microbiota in Health and Disease: In Vitro Modeling of Host‐Microbe Interactions at the Aerobe‐Anaerobe Interphase of the Human Gut.” Anaerobe 44: 3–12. 10.1016/j.anaerobe.2017.01.001. 28062270

[fsn370159-bib-0169] Wallace, M. , T. Hicks , Y. Z. Khimyak , and J. Angulo . 2019. “Self‐Correcting Method for the Measurement of Free Calcium and Magnesium Concentrations by ^1^H NMR.” Analytical Chemistry 91, no. 22: 14442–14450. 31613090 10.1021/acs.analchem.9b03008

[fsn370159-bib-0132] Wan, M. L. , K. H. Ling , H. El‐Nezami , and M. F. Wang . 2019. “Influence of Functional Food Components on Gut Health.” Critical Reviews in Food Science and Nutrition 59, no. 12: 1927–1936. 10.1080/10408398.2018.1433629. 29381385

[fsn370159-bib-0150] Wei, X. , H. Wu , Z. Wang , et al. 2023. “Rumen‐Protected Lysine Supplementation Improved Amino Acid Balance, Nitrogen Utilization and Altered Hindgut Microbiota of Dairy Cows.” Animal Nutrition 15: 320–331. 10.1016/j.aninu.2023.08.001. 38053803 PMC10694044

[fsn370159-bib-0134] Wu, Y. , C. P. Zhu , Y. Zhang , Y. Li , and J. R. Sun . 2019. “Immunomodulatory and Antioxidant Effects of Pomegranate Peel Polysaccharides on Immunosuppressed Mice.” International Journal of Biological Macromolecules 137: 504–511. 31229542 10.1016/j.ijbiomac.2019.06.139

[fsn370159-bib-0159] Xiong, J. , F. Chen , J. Zhang , et al. 2022a. “Occurrence of Aflatoxin M1 in Three Types of Milk from Xinjiang, China, and the Risk of Exposure for Milk Consumers in Different Age‐Sex Groups.” Food 11, no. 23: 3922. 10.3390/foods11233922. PMC9738243 36496730

[fsn370159-bib-0160] Xiong, J. , D. Wen , H. Zhou , et al. 2022b. “Occurrence of Aflatoxin M1 in Yogurt and Milk in Central‐Eastern China and the Risk of Exposure in Milk Consumers.” Food Control 137: 108928. 10.1016/j.foodcont.2022.108928.

[fsn370159-bib-0155] Yan, Y. , B. Li , Q. Gao , et al. 2025. “Intestine‐Decipher Engineered Capsules Protect Against Sepsis‐induced Intestinal Injury via Broad‐spectrum Anti‐inflammation and Parthanatos Inhibition.” Advanced Science 12, no. 10: 2412799. 10.1002/advs.202412799. 39836501 PMC11904959

[fsn370159-bib-0137] Yoo, J. Y. , M. Groer , S. V. O. Dutra , A. Sarkar , and D. I. McSkimming . 2020. “Gut Microbiota and Immune System Interactions.” Microorganisms 8, no. 10: 1587. 10.3390/microorganisms8101587. 33076307 PMC7602490

[fsn370159-bib-0138] Yu, Z. , G. Liu , S. Li , et al. 2024. “Effects of Fermented Pomegranate Peel Polyphenols on the Growth Performance, Immune Response, Hepatopancreas Health and Disease Resistance in White Shrimp (Litopenaeus Vannamei).” Aquaculture Nutrition 20: 772. 10.1155/anu/9966772 PMC11617047 39633958

[fsn370159-bib-0139] Yuen, B. 2021. Effects of Inulin, Fructooligosaccharide, and Breadfruit Fiber on Biofilm Formation, Growth, and Gastrointestinal Survival of Probiotic Yeast and Bacteria (Master's thesis). University of Hawai'i.

[fsn370159-bib-0140] Zang, E. , L. Jiang , H. Cui , et al. 2023. “Only Plant‐Based Food Additives: An Overview on Application, Safety, and Key Challenges in the Food Industry.” Food Reviews International 39, no. 8: 5132–5163. 10.1080/87559129.2022.2062764.

[fsn370159-bib-0151] Zeng, M. , Y. Zou , Z. Shi , et al. 2024. “A Broad‐Spectrum Broth Rapidly and Completely Repairing the Sublethal Injuries of Escherichia coli Caused by Freezing and Lactic Acid Alone or in Combination for Accurate Enumeration.” LWT 201: 116219. 10.1016/j.lwt.2024.116219.

[fsn370159-bib-0141] Zhang, L. , H. Zhan , W. Xu , S. Yan , and S. C. Ng . 2021. “The Role of Gut Mycobiome in Health and Diseases.” Therapeutic Advances in Gastroenterology 14: 175628. 10.1177/17562848211047130 PMC8474302 34589139

[fsn370159-bib-0142] Zhang, N. , Z. Ju , and T. Zuo . 2018. “Time for Food: The Impact of Diet on Gut Microbiota and Human Health.” Nutrition 51: 80–85. 29621737 10.1016/j.nut.2017.12.005

[fsn370159-bib-0149] Zhang, Y. , X. Zhang , D. Cao , et al. 2024. “Integrated Multi‐Omics Reveals the Relationship Between Growth Performance, Rumen Microbes and Metabolic Status of Hu Sheep with Different Residual Feed Intakes.” Animal Nutrition 18: 284–295. 10.1016/j.aninu.2024.04.021. 39281047 PMC11402313

[fsn370159-bib-0176] Zhao, D. , J. E. Simon , and Q. Wu . 2020. “A Critical Review on Grape Polyphenols for Neuroprotection: Strategies to Enhance Bioefficacy.” Critical Reviews in Food Science and Nutrition 60, no. 4: 597–625. 30614258 10.1080/10408398.2018.1546668

[fsn370159-bib-0143] Zheng, Z. , and B. Wang . 2021. “The Gut‐Liver Axis in Health and Disease: The Role of Gut Microbiota‐Derived Signals in Liver Injury and Regeneration.” Frontiers in Immunology 12: 775526. 34956204 10.3389/fimmu.2021.775526 PMC8703161

[fsn370159-bib-0152] Zhu, Z. , Y. Gu , C. Zeng , et al. 2022. “Olanzapine‐Induced Lipid Disturbances: A Potential Mechanism Through the Gut Microbiota‐Brain Axis.” Frontiers in Pharmacology 13: 897926. 10.3389/fphar.2022.897926. 35991866 PMC9388751

[fsn370159-bib-0166] Ziegler, D. , N. Papanas , O. Schnell , et al. 2021. “Current Concepts in the Management of Diabetic Polyneuropathy.” Journal of Diabetes Investigation 12, no. 4: 464–475. 32918837 10.1111/jdi.13401 PMC8015839

